# Cost-effectiveness of PCV13 vaccination in Belgian adults aged 65-84 years at elevated risk of pneumococcal infection

**DOI:** 10.1371/journal.pone.0199427

**Published:** 2018-07-06

**Authors:** Sophie Marbaix, Willy E. Peetermans, Jan Verhaegen, Lieven Annemans, Reiko Sato, Annick Mignon, Mark Atwood, Derek Weycker

**Affiliations:** 1 Pfizer NV/SA, Brussels, Belgium; 2 Department of Internal Medicine, University Hospital Leuven, Leuven, Belgium; 3 Department of Microbiology, University Hospital Leuven, Leuven, Belgium; 4 Ghent University, Ghent, Belgium; 5 Pfizer Inc., Collegeville, PA, United States of America; 6 Policy Analysis Inc. (PAI), Brookline, MA, United States of America; Defense Threat Reduction Agency, UNITED STATES

## Abstract

**Background:**

The Belgian Superior Health Council (SHC) recently added a 13-valent pneumococcal conjugate vaccine (PCV13) to its recommendations for adult pneumococcal vaccination. This study addresses the policy question regarding whether a single dose of PCV13 should be reimbursed among Belgian adults aged 65–84 years with chronic comorbidities (“moderate-risk”) or immunosuppression (“high-risk”).

**Methods:**

A cohort model was developed to project lifetime risks, consequences, and costs of invasive pneumococcal disease (IPD) and pneumococcal community-acquired pneumonia (CAP). Parameter values were estimated using published literature and available databases, and were reviewed by Belgian experts. PCV13 effectiveness was assumed to be durable during the first 5 years following receipt, and to progressively decline thereafter with 15 years protection. The Belgian National Health Insurance perspective was employed.

**Results:**

Use of PCV13 (vs. no vaccine) in moderate/high-risk persons aged 65–84 years (n = 861,467; 58% vaccination coverage) would be expected to prevent 527 cases of IPD, 1,744 cases of pneumococcal CAP and 176 pneumococcal-related deaths, and reduce medical care costs by €20.1 million. Vaccination costs, however, would increase by €36.9 million and thus total overall costs would increase by €16.8 million. Cost per QALY gained was €17,126. In probabilistic sensitivity analyses, use of PCV13 was cost-effective in 97% of 1,000 simulations.

**Conclusions:**

Reimbursement of PCV13 in moderate/high-risk Belgian adults aged 65–84 years would be cost-effective from the Belgian healthcare perspective.

## Introduction

*Streptococcus pneumoniae* causes significant morbidity and mortality worldwide in both children and adults. Invasive pneumococcal disease (IPD)—including bacteremia and meningitis—is the most serious manifestation because of its high mortality rate, but is relatively uncommon. Nonbacteremic/noninvasive pneumococcal community-acquired pneumonia (CAP) has a much higher incidence rate and also is associated with significant morbidity, mortality, and healthcare costs. While widespread childhood use of pneumococcal conjugate vaccines has reduced the incidence of invasive pneumococcal disease, the burden of both invasive and noninvasive disease in older adults remains substantial [1–4].

Routine immunization is the principal means of preventing pneumococcal infection, and while a 23-valent pneumococcal polysaccharide vaccine (PPV23) has been available in Belgium since 1995, it has been infrequently administered among older adults [5]. Considering the remaining vaccine-preventable disease burden, in April 2015, the Belgian Superior Health Council (SHC) added a 13-valent pneumococcal conjugate vaccine (PCV13) to its recommendations for adult pneumococcal vaccination. Specifically, the SHC now recommends use of PCV13—followed by PPV23—in persons aged 18–84 years with impaired immune systems (“high-risk”), persons aged 50–84 years with chronic comorbidities (“moderate-risk”), and “low-risk” persons aged 65–84 years who are healthy.

The recommendation was based in large part on findings from the Community-Acquired Pneumonia Immunization Trial in Adults (CAPiTA), in which PCV13 demonstrated protection against community-acquired pneumococcal pneumonia (pneumococcal CAP) and IPD [6,7]. This trial, which followed 84,496 low-risk and moderate-risk adults aged ≥65 years in the Netherlands for an average of four years, is one of the largest adult vaccine efficacy trials ever conducted. During the CAPiTA study, PCV13 demonstrated statistically significant reductions in first episodes of vaccine-type pneumococcal CAP (45.6% [95%CI: 21.8–62.5]), vaccine-type nonbacteremic/noninvasive pneumococcal CAP (45.0% [95%CI: 14.2–65.3]), and vaccine-type IPD (75.0% [95%CI: 41.4–90.8]). The duration of protection extended over the 4-year study. A *post-hoc* analysis of data from CAPiTA found similar reductions in the first episode of vaccine-type pneumococcal CAP among at-risk subjects (40.3% [95%CI: 11.4–60.2])—which included adults with lung disease, heart disease, liver disease, diabetes, or asthma (self-reported)—compared with the overall study population, suggesting similar efficacy against vaccine-type CAP in at-risk adults as in the general population [8].

While a single dose of PCV13 is now recommended for many age- and risk-specific adult subgroups in Belgium, the economic implications of vaccine reimbursement in these patient populations are unknown. Blommaert et al. have investigated the cost-effectiveness of PCV13 in Belgian adults aged >50 years, on an overall basis (i.e., irrespective of risk profile) as well as within the healthy subgroup, and concluded that PCV13 vaccination is unlikely to be cost-effective [9,10]. Their findings, however, are not generalizable to adults who are at higher risk of pneumococcal disease and its consequences (namely, the moderate- and high-risk elderly populations), for whom the clinical benefit—and thus economic value—of PCV13 may be substantially greater. We therefore undertook a pharmacoeconomic assessment to address the policy question regarding whether a single dose of PCV13 should be reimbursed among Belgian adults aged 65–84 years who are at moderate-risk or high-risk of pneumococcal infection and its complications.

## Materials and methods

### Model description

**Fig 1:**
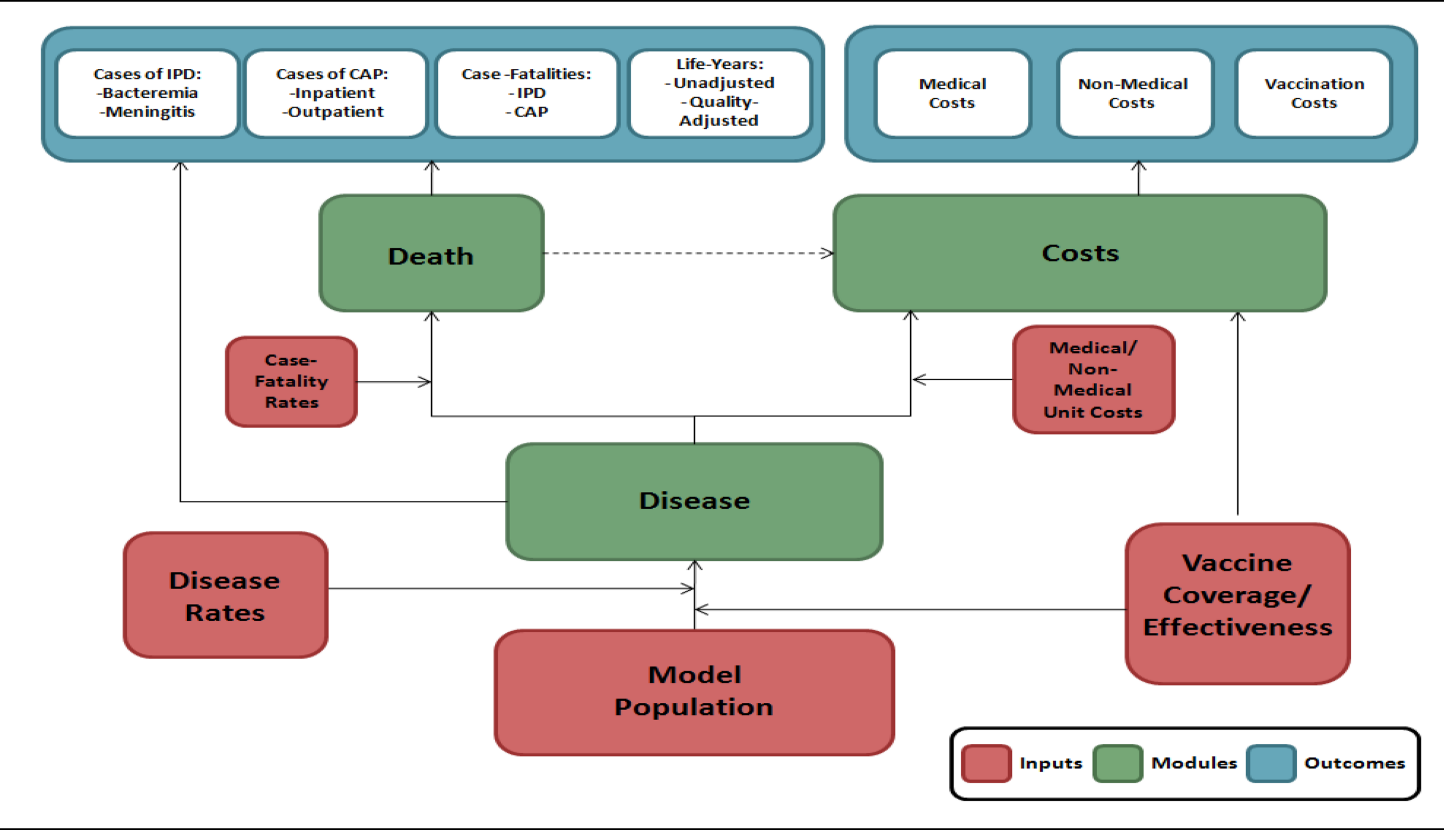
Model schematic.

The model utilizes a deterministic cohort framework and a Markov-type process to depict expected lifetime risks, consequences, and costs of IPD and pneumococcal CAP, as well as the expected impact of PCV13 vaccination, in Belgian adults (Fig 1). Both IPD and pneumococcal CAP were included in the model because of their impact among the elderly: IPD is the most serious manifestation of S. pneumoniae because of its high mortality rate, and pneumococcal CAP, while also associated with significant morbidity and mortality, is much more common. Moreover, PCV13 has been found to be effective in reducing the risk of these events in the elderly (i.e., in CAPiTA) and thus may positively impact health and quality of life. The materials and methods of this study—including model structure and model parameters—are, in many cases, similar to those employed in a prior economic/public health evaluation of PCV13 in the United States that was conducted by some of the study investigators [11].

The model cohort is characterized in terms of age and risk profile (low-risk, moderate-risk, high-risk). Low-risk includes persons who are immunocompetent without chronic comorbidities, moderate-risk includes persons who are immunocompetent with ≥1 chronic comorbidity, and high-risk includes persons who are immunocompromised (note: splenectomised patients, who are included in the SHC recommendation, were not explicitly considered in the high-risk group due to limitations of available data). Low-risk persons may transition to the moderate-risk or high-risk groups during the modeling horizon based on the probability of newly developing a chronic comorbidity or immunocompromising condition, respectively; moderate-risk persons may similarly transition to the high-risk group. Persons in the model cohort may receive PCV13 or no vaccine at model entry; vaccination coverage may vary by age and risk profile. No vaccine was selected as the comparator because PPV23 is infrequently used among Belgian adults (e.g., in 2013, only 10% of persons aged ≥65 years indicated that they had been vaccinated during the prior 5-year period) and PCV13 will not replace PPV23 (cfr SHC recommendation). The current situation considering 10% vaccination with PPV23 and 90% no vaccination will be used as comparator in a scenario analysis [12].

Expected clinical outcomes and economic costs are estimated for the model cohort on an annual basis, based on age, risk profile, vaccination status, and time since vaccination. IPD is stratified by condition (bacteremia vs. meningitis), and pneumococcal CAP is stratified by setting of care (hospitalized vs. ambulatory). Persons vaccinated at model entry may be at lower risk of future IPD and pneumococcal CAP; the magnitude of vaccine-associated risk reduction depends on clinical presentation (i.e., IPD or pneumococcal CAP), as well as time since vaccination, age, and risk profile. Risk of death from IPD, hospitalized CAP, and all other causes (general population mortality less deaths due to IPD and pneumococcal CAP) depends upon age and risk profile.

Expected costs of medical treatment for IPD and pneumococcal CAP are generated based on event risks and unit costs in relation to the setting of care (i.e., inpatient vs. outpatient), age, and risk profile. Costs of vaccination—including vaccine and vaccine administration—are tallied at model entry. Clinical outcomes and economic costs are projected over the modelling horizon for the model cohort under the vaccination strategies considered, and include: cases of IPD (bacteremia and meningitis) and pneumococcal CAP (hospitalized and outpatient [i.e., ambulatory]); deaths due to IPD and CAP; life-years (unadjusted and quality-adjusted); costs of medical treatment for IPD and CAP; and costs of vaccination.

### Model estimation

Rates of IPD and pneumococcal CAP (adjusted, as warranted, to reflect assumed herd effects from the pediatric vaccination program in Belgium), case-fatality rates, and disease-specific episodic costs—by age and risk profile (moderate-risk, high-risk)—were estimated based on age-specific values from Belgium sources, age-specific relative rates/costs by risk profile from Belgium, UK and US sources, and age-specific population weights by risk profile from Belgium sources. The incidence and mortality values reflect a conservative approach [10]. A detailed explanation of methods and sources that were employed to estimate these parameters as well as vaccine effectiveness, vaccination costs, and health-state utilities is available in the S1 File. A summary of methods and sources used in the estimation of key variables is set forth below, and parameter values for key variables are reported in Table 1 and Table 2.

**Table 1:** Estimates of population size, disease rates, case-fatality rates, and associated costs*.

	65–74 Years		75–84 Years		≥85 Years	
	Moderate	High	Moderate	High	Moderate	High
No. of Belgian Adults	420,774	44,181	365,901	30,610	153,348	6,495
Annual Disease Incidence (per 100K)						
Bacteremia	37.4	82.0	48.0	105.2	107.0	234.5
Meningitis	1.69	3.71	2.18	4.78	5.03	11.02
Pneumococcal CAP						
Inpatient	129	439	186	599	546	1,733
Outpatient	89	305	134	433	207	666
Annual Case-Fatality (per 100)						
Bacteremia	14.0	14.0	17.6	17.6	24.2	24.2
Meningitis	12.8	12.8	24.0	24.0	53.9	53.9
Pneumococcal CAP						
Inpatient	3.9	3.9	5.6	5.6	8.6	8.6
Outpatient	1.9	1.9	1.8	1.8	1.8	1.8
Utilities						
General Population Utility	0.7962	0.6001	0.7162	0.5490	0.6238	0.5876
Annual Disutility due to Disease						
Bacteremia	0.1759	0.1320	0.1758	0.1354	0.1741	0.1741
Meningitis	0.1759	0.1320	0.1758	0.1354	0.1741	0.1741
Pneumococcal CAP						
Inpatient	0.0717	0.0537	0.0716	0.0551	0.0709	0.0709
Outpatient	0.0066	0.0049	0.0047	0.0036	0.0027	0.0027
Medical Care Costs (per case)						
Requiring Inpatient Care						
Bacteremia	€ 15,439	€ 12,338	€ 16,658	€ 13,313	€ 16,543	€ 13,221
Meningitis	€ 11,279	€ 9,014	€ 10,932	€ 8,737	€ 10,857	€ 8,676
Pneumococcal CAP	€ 8,501	€ 9,359	€ 16,073	€ 17,695	€ 15,482	€ 17,044
Requiring Outpatient Care Only						
Pneumococcal CAP	€ 867	€ 985	€ 864	€ 982	€ 866	€ 984
Vaccination (per person)						
PCV13	€ 63.64	€ 63.64	€ 63.64	€ 63.64	€ 63.64	€ 63.64
Administration	€ 10.24	€ 10.24	€ 10.24	€ 10.24	€ 10.24	€ 10.24

*Methods and sources used in estimating parameter values set forth in S1 File

**Table 2:** Effectiveness of PCV13*.

	VE-PCV13, by No. of Years Since Receipt of Vaccine				
	1	5	10	15	20
IPD (due to vaccine serotypes**)					
Age/Risk Profile					
65–74 years					
Moderate-Risk	84%	79%	71%	64%	0%
High-Risk	65%	62%	56%	50%	0%
75–84 years					
Moderate-Risk	70%	66%	60%	54%	0%
High-Risk	55%	52%	47%	42%	0%
Pneumococcal CAP (due to vaccine serotypes**)					
Age/Risk Profile					
65–74 Years					
Moderate-Risk	41%	39%	35%	32%	0%
High-Risk	27%	25%	23%	21%	0%
75–84 Years					
Moderate-Risk	39%	37%	33%	30%	0%
High-Risk	25%	24%	22%	19%	0%

*Methods and sources used in estimating parameter values set forth in S1 File

**PCV13 serotype coverage (year 1): IPD, 30.9%; pneumococcal CAP, 32.9%

#### Rates of IPD

Age-specific rates of bacteraemia and meningitis were based on 2015 surveillance data from the Belgian National Reference Centre, and were extrapolated to the Belgian adult population [10]. Age-specific values were allocated across risk groups based on estimated rate ratios from a study of the impact of underlying medical conditions on the risk of IPD in England and age/risk-specific Belgian population weights [13]. Baseline rates of bacteremia and meningitis (2015) were adjusted (i.e., reduced) to account for residual indirect effects due to widespread use of PCV13 in children up to 2015 (mid-2015 in Flanders) before the switch to PCV10 in 2016. The percentage of IPD due to PCV13 serotypes (30.9%) was assumed to persist throughout the modeling horizon.

#### Rates of pneumococcal CAP

Age- and risk-specific rates of all-cause outpatient pneumonia were estimated using 2013 data from the INTEGO Database, which was assumed to be representative of the Belgian population [10,14]. Episodes of outpatient pneumonia were identified using code R81 (“Pneumonia”) from the international classification of primary care (ICPC); 15% of outpatient CAP was assumed to be pneumococcal.

Age-specific rates of pneumococcal CAP requiring inpatient care (i.e., hospitalized CAP) were estimated using data from the KCE report [10]. Age-specific hospitalized CAP values were allocated across risk groups using the outpatient pneumonia rate ratios. In line with the KCE report [10], the indirect effects from childhood PCV13 use were assumed to be the same for pneumococcal non-invasive CAP and IPD. The percentage of pneumococcal CAP due to PCV13 serotypes (32.9%) was assumed to persist throughout the modeling horizon.

#### VE-PCV13 vs. VT-IPD

Effectiveness of PCV13 against vaccine-type IPD for immunocompetent (i.e., moderate-risk) persons with chronic comorbidities was based on the estimated efficacy of PCV13 against vaccine-type IPD among subjects in the per-protocol population of the CAPiTA study [6]. The impact of age at vaccination on the VE was based on a post-hoc analysis of CAPiTA [15]. For subjects in the high-risk group, PCV13 VE against VT-IPD was assumed to be 22% lower than corresponding values for the low-/moderate-risk populations, based on the relative difference in VE observed in pneumococcal vaccination of children with and without HIV [16].

#### VE-PCV13 vs VT-CAP

Effectiveness of PCV13 against VT-CAP for immunocompetent persons with chronic comorbidities (i.e., moderate-risk persons) was based on the estimated efficacy of PCV13 against vaccine-type CAP among subjects in the per-protocol population of a post-hoc analysis of the CAPiTA study [17]. In the absence of information, similar VE was assumed for outpatient CAP; the same approach was considered by Blommaert et al. [10]. Change in VE-PCV13 with age was estimated based on a previous study (see S1 File) [11]. For subjects in the high-risk group, VE-PCV13 against VT-CAP was assumed to be 35% lower than corresponding values for the moderate-risk population, based on the relative difference in VE observed in pneumococcal vaccination of children with and without HIV [16].

#### VE-PCV13 waning

VE was considered stable during the first 5 years following vaccination [6], and thereafter assumed to wane annually at a rate of 5% during years 6–10, 10% annually during years 11–15, and no efficacy was assumed from year 16 onwards. This approach is in line with that of CAPiTA investigators [18]. Based on CAPiTA, PCV13 vaccination was assumed not to have serious adverse effects [6].

#### Medical-care and vaccination costs

Medical-care costs were derived from the database of a large sickfund and interviews [9,19,20], and were reviewed and informally validated by a panel of four independent Belgian experts. All IPD cases were assumed to require hospitalization, and corresponding costs were not limited to those incurred while in hospital; the same approach was used for pneumonia requiring inpatient care. Age-specific cost estimates were based on data from a national face-to-face survey by members of the Sickfunds for IPD cases and CAP cases with pneumococcus isolation, and were allocated across risk groups based on multiple factors as derived from a published source [21]. Cost of outpatient pneumonia for persons aged ≥65 years was estimated to be €848 based on the same sources of information [9,19,20], and was allocated across risk groups as described above [21].

The reimbursed price of PCV13 was assumed to be €63.64 per dose, based on the public price (€75.44) [22]. Because PCV13 would be typically administered to this target population (immunocompetent with chronic-comorbidities or immunocompromised patients) during a routine GP visit, or in combination with a visit for influenza vaccination, the cost of vaccine administration was assumed equal to 50% of the cost of a GP visit (€20.48) [23]. The same approach was considered in the KCE report [10]. All costs were expressed at 2016 prices.

### Analyses

#### Base case

Clinical outcomes and economic costs were projected over remaining years of life for moderate/high-risk Belgian adults aged 65–84 years at model entry, under two alternative vaccination strategies: (1) use of PCV13 at model entry (58% vaccination coverage level); and (2) no vaccination at model entry. Analyses were conducted from the perspective of the Belgian healthcare public payer; accordingly, only direct costs associated with the provision of medical care for IPD and pneumococcal CAP, and the costs of vaccination, were considered. Results were standardized to the Belgian population of interest based on age- and risk-specific weights. Medical-care costs were discounted at 3% annually, and life-years were discounted at 1.5% annually [20].

The cost-effectiveness of PCV13 (vs. no vaccination) was calculated in terms of the cost per life-year gained and cost per QALY gained, respectively, which are derived as the ratio of the difference in total costs to the difference in total life-years/QALYs. In Belgium, a willingness-to-pay threshold—below which interventions are considered cost-effective—has not been formalized. However, the Belgian Health Care Knowledge Centre (KCE) has recently used three alternative thresholds in health technology assessments of pneumococcal vaccination in the elderly, namely 35,000€, 70,000€, and 105,000€, which correspond to 1x, 2x, and 3x the gross domestic product (GDP) per inhabitant [10].

#### Sensitivity

One-way deterministic sensitivity analyses were undertaken to evaluate the potential impact of parameter value uncertainty on study results for moderate/high-risk adults aged 65–84 years. In these analyses, key model parameter values were varied ±25% of their base case levels (unless otherwise noted below), each in turn, including: rates of ambulant and hospitalized pneumococcal CAP; age-specific utility values; case-fatality rates; vaccine effectiveness (vs. IPD and pneumococcal CAP); vaccine effectiveness (vs. IPD and pneumococcal CAP) for high-risk adults relative to immunocompetent adults; medical-care costs; and herd effect for vaccine-type IPD and pneumococcal CAP (varied from 0–100%). Probabilistic sensitivity analysis (n = 1,000 replications) was employed to account for uncertainty surrounding disease rates and costs, case-fatality rates, and vaccine effectiveness and other key model parameters in estimation of clinical outcomes, economic costs, and incremental cost-effectiveness ratios.

#### Scenario

In addition, scenario analyses were conducted to evaluate the impact of methodological uncertainty on study results for moderate/high-risk adults aged 65–84 years. In these analyses, alternative data sources and/or assumptions were employed in estimating key variables, including: current situation as comparator (PPV23 vaccine coverage of 10%/12.9% persons aged 65–74 years and 75–84 years, respectively); higher case-fatality rates (based on Blommaert 2016a), PCV13 serotype coverage against pneumococcal CAP (±25% base case values), higher rate of waning for PCV13 (25% annual logistic waning with 50% initial effectiveness achieved in year 10), vaccine coverage (33% and 83%, respectively), discount rate (0% and 5%, respectively), and population comprising persons aged 65 years only. Scenario analyses focusing on moderate-risk Belgian adults aged 65–84 years and high-risk Belgian adults aged 65–84 years, respectively, were also conducted.

## Results

### Base case

Without vaccination, the expected lifetime numbers of total cases of disease among moderate-risk and high-risk Belgian adults aged 65–84 years (n = 861,467) would be: IPD, 7,352; hospitalized pneumococcal CAP, 33,662; and outpatient pneumococcal CAP, 17,617 (Table 3). Expected lifetime medical-care costs to treat these events would total €472.1 million. With use of PCV13 among 58% of moderate and high-risk Belgian adults aged 65–84 years (n = 861,467), the expected lifetime numbers of cases of disease would total: IPD, 6,825; hospitalized pneumococcal CAP, 32,588; and outpatient pneumococcal CAP, 16,947. Expected lifetime medical-care costs would total €452.0 million, and vaccination costs, €36.9 million.

**Table 3:** Cost-effectiveness/utility of PCV13 versus no routine vaccination in moderate/high-risk Belgian adults aged 65–84 years.

	No Vaccine	PCV13	Difference
Population-Level Results			
No. of Cases			
IPD	7,352	6,825	-527
Pneumococcal CAP			
Hospitalized	33,662	32,588	-1,074
Outpatient	17,617	16,947	-669
No. of Deaths	4,279	4,104	-176
Total Costs (in million)			
Medical Care	472.13	452.03	-20.10
Vaccination	0.00	36.91	36.91
Total			
Medical + Vaccination	472.13	488.95	16.82
Life-Years (discounted)	9,590,018	9,591,268	1,251
Quality-Adjusted Life-Years (discounted)	6,724,131	6,725,113	982
Cost-Effectiveness			
Cost per Life-Year Gained			€ 13,444
Cost per Quality-Adjusted Life-Year Gained			€ 17,126

On balance, therefore, use of PCV13—as described above—would reduce lifetime cases of IPD by 527, hospitalized pneumococcal CAP by 1,074, and outpatient pneumococcal CAP by 669; disease-related deaths by 176; and medical-care costs (excluding vaccination costs) by €20.1 million. Medical-care and vaccination costs (aggregated) would increase by €16.8 million. Cost per QALY gained (from the health care perspective) was €17,126.

### Sensitivity and scenario analyses

**Fig 2:**
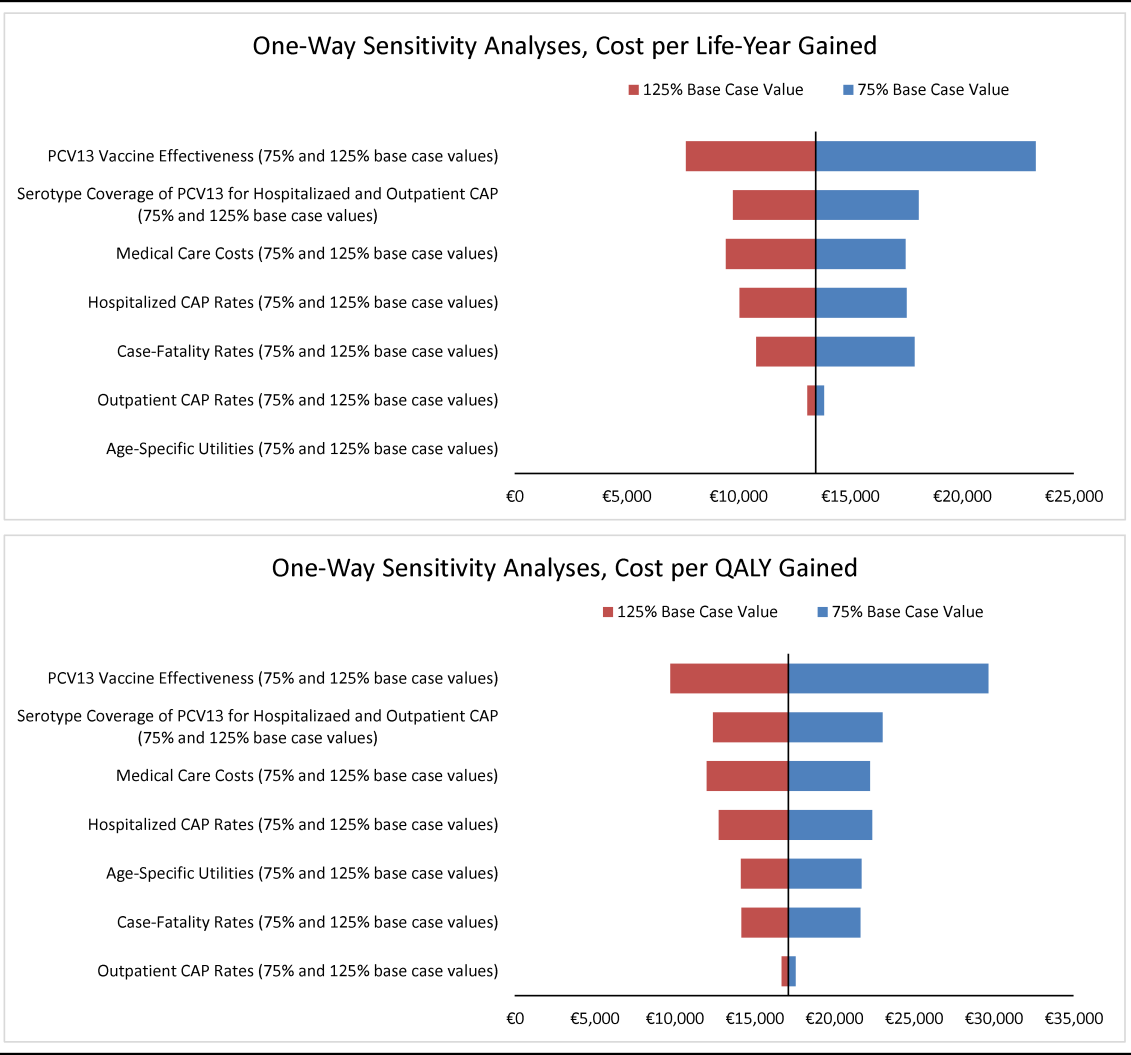
One-way deterministic sensitivity analyses on cost-effectiveness/utility of PCV13 versus no routine vaccination in moderate/high-risk Belgian adults aged 65–84 years.

In all of the aforementioned one-way deterministic sensitivity analyses, use of PCV13 among moderate-risk and high-risk Belgian adults aged 65–84 years was found to be cost-effective versus no routine vaccination (Fig 2). Cost-effectiveness was most sensitive to variation in the effectiveness of PCV13; when assuming values equal to 75% of base case, the ratio increases from €17,126 to €29,661. When assuming 75% of values for—each in turn—PCV13 serotype coverage in pneumococcal CAP, medical-care costs, hospitalized pneumococcal CAP rates, utilities, case-fatality rates, and outpatient pneumococcal CAP rates, respectively, cost-effectiveness ratios ranged from €21,638 to €23,022.

In the scenario analysis comparing use of PCV13 (58% coverage) versus use of PPV23 (10% coverage in persons aged 65–74 years, 12.9% coverage in persons aged 75–84 years) among moderate/high-risk Belgian adults aged 65–84 years, use of PCV13 was found to be cost-effective. Similarly, in the scenario analysis comparing use of PCV13 versus no vaccine in a cohort of moderate/high-risk Belgian adults aged 65 years, and those analyses focusing (separately) on moderate-risk Belgian adults aged 65–84 years and high-risk Belgian adults aged 65–84 years, use of PCV13 was found to be cost-effective. Results from all sensitivity analyses and all scenario analyses are set forth in the S1 File.

**Fig 3:**
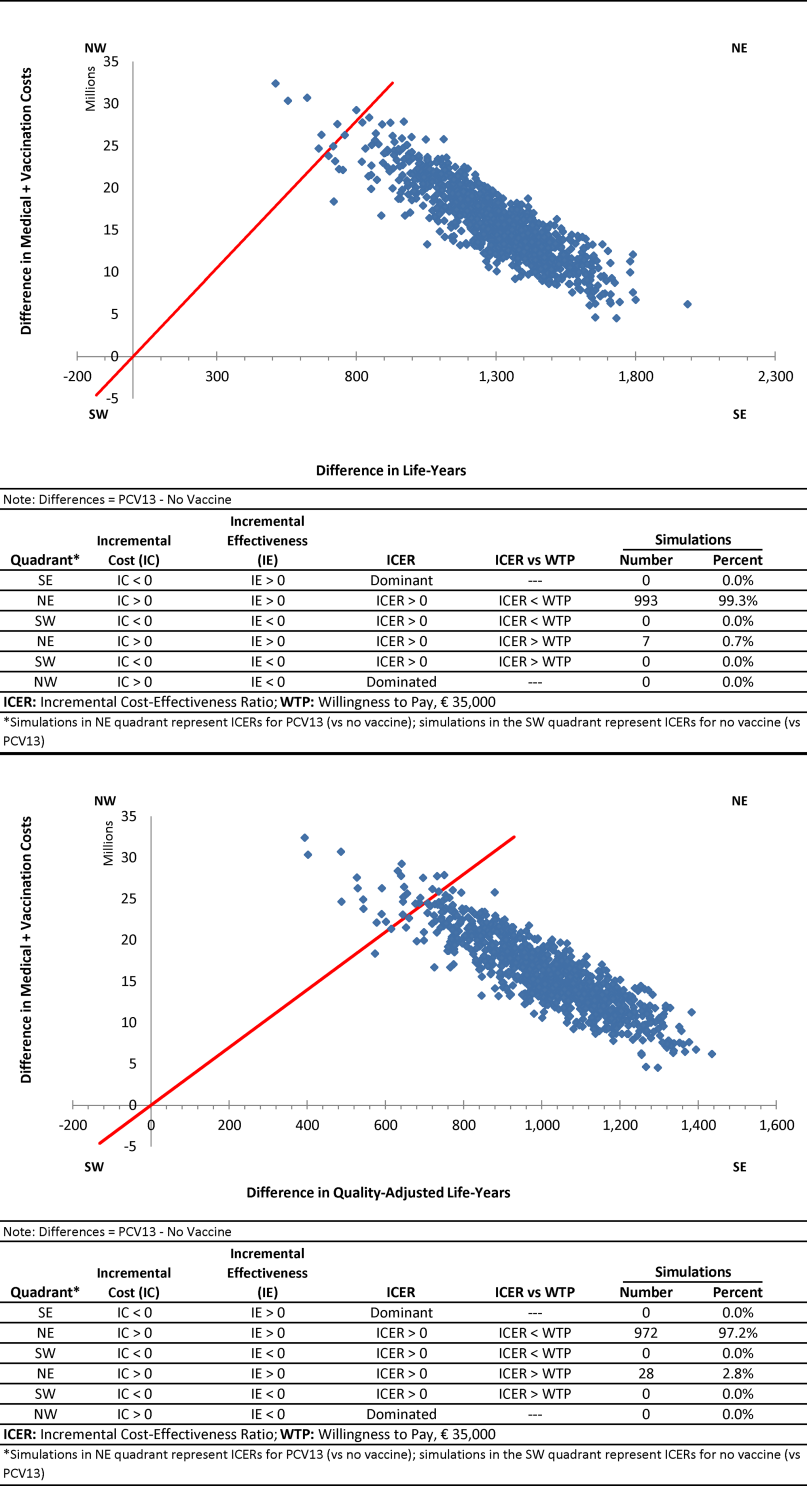
Scatterplots for cost-effectiveness/utility of PCV13 versus no routine vaccination in moderate/high-risk Belgian adults aged 65–84 years.

In the probabilistic sensitivity analysis, all of the 1,000 replications generated estimates of incremental cost per LY/QALY gained that were located in the northeast quadrant of the scatterplot, and thus projected higher costs and higher LY/QALY with use of PCV13 versus no routine vaccination (Fig 3). In this same analysis, the incremental cost per LY/QALY gained was less than the assumed maximum willingness to pay for 99%/97% of the 1,000 replications, respectively.

## Discussion

Since April 2015, the SHC in Belgium has included PCV13 in their recommendations for pneumococcal vaccination among adults of various ages and risk profiles. While Blommaert et al. [9,10] have analyzed the cost-effectiveness of PCV13 in a general adult population aged >50 years, cost-effectiveness within risk groups has not been previously investigated in Belgium settings. We therefore considered the policy question regarding whether a single dose of PCV13 should be reimbursed among Belgian adults aged 65–84 years who are at moderate-risk or high-risk of pneumococcal infection and its complications.

Our findings suggest that, under conservative assumptions concerning disease burden and reasonable assumptions concerning vaccine effectiveness and costs, implementation of a strategy targeting Belgian moderate-risk and high-risk adults aged 65–84 years for vaccination with PCV13 would reduce the numbers of cases of pneumococcal disease and pneumococcal-related deaths, would—on an overall basis—partly offset the cost of the vaccination from the healthcare public system perspective, and would be a cost-effective use of scarce healthcare resources. Notwithstanding differences in model structure, model population, model estimation, and vaccination strategies, our conclusions are largely consistent with those from several recent evaluations in which adult PCV13 use (mainly persons aged ≥65 years) was found to have a reasonable cost-effectiveness profile based on current disease epidemiology [18,24–26]. In the three recent evaluations in which PCV13 was found not to be cost-effective, two considered different populations (namely a general adult population aged >50 years in Belgium, without taking into consideration subgroup analysis by risk of pneumococcal infection), while the other study assumed disease burden to be considerably lower than current levels based on future projections of indirect effects from the pediatric program [9,10,27]. Our findings also suggest that the clinical benefits and economic value of PCV13 may be substantially greater within the moderate-risk and high-risk elderly Belgian populations relative to their healthy counterparts. In our study, PCV13 (vs. no vaccination) was reported to cost €17,126 per QALY gained among moderate/high-risk Belgian adults aged 65–84 years. At a minimum, these findings support the SHC recommendation and vaccine reimbursement for use of PCV13 in persons aged 65–84 years with impaired immune systems and persons aged 65–84 years with chronic comorbidities.

The results of analyses described herein are based on data and assumptions specific to the vaccination environment (past, current, and future [projected]) in Belgium, and thus reflect the recommendations and use of PCV13, PPV23, and PCV10 in that setting. While findings were projected assuming conservative values for several key model parameters, and were robust when varying key model parameters in sensitivity analyses (including higher waning of PCV13 effectiveness and herd effect from PCV10 use), caution should be exercised in generalizing the results of this study to other countries in which vaccine recommendations and use may be different among children and adults. The results of this assessment are based on certain assumptions regarding the level and durability of PCV13 effectiveness among adults, and the level and persistence of indirect vaccination effects from the Belgian pediatric program, and thus are limited to the scenarios considered herein until proven otherwise. To the extent new data become available, study results will require updating.

A modelling approach was chosen because, for several reasons, a trial-based (i.e., CAPiTA-based) pharmacoeconomic evaluation focusing on the population of interest was not possible. CAPiTA enrolled only subjects in the Netherlands, and the epidemiology of pneumococcal disease and the associated use of healthcare resources may be different in important aspects in Belgium. CAPiTA enrolled only subjects who were immunocompetent, and thus high-risk persons were not included in the trial population (i.e., at the time of trial baseline). Follow-up in CAPiTA extended for only 4–5 years (on average), and thus the long-term/life-time benefits of vaccination were not captured. Moreover, we note that the modelling approach described herein—in terms of the structure of the model and the methods of estimation—is similar to that employed in other recently published economic evaluations of alternative strategies for pneumococcal vaccination in other countries [11,20,28–33].

We believe that the major limitation of this assessment is the uncertainty concerning some of the parameter estimates. Perhaps the area of greatest uncertainty concerns the assumed effectiveness of vaccination with PCV13, which was based primarily on data from the CAPiTA trial [6,34]. Effectiveness of PCV13 against all-cause pneumococcal CAP was derived based on the estimated efficacy against vaccine-type, non-bacteremic/non-invasive CAP (40%) among at-risk persons from CAPiTA, and the percentage of all-cause pneumococcal CAP assumed to be attributable to PCV13 serotypes based on extrapolation from PCV13 serotypes in IPD (32.9%) [6,10,18]. Due to the impact of this parameter and the lack of local data, this value was subjected to sensitivity analysis. Similarly, data on the durability of PCV13 effectiveness over time were not available from CAPiTA (i.e., beyond the follow-up period [mean, 4+ years]), and thus PCV13 effectiveness (i.e., after the initial 5 years of the modelling horizon) was assumed to wane annually at 5% during years 6–10, at 10% annually during years 11–15, and to be 0% beginning in year 16, consistent with methods employed in another economic evaluation [18]. Finally, because CAPiTA enrolled only immunocompetent subjects, and thus data from this trial on PCV13 effectiveness were not directly applicable to high-risk persons, vaccine effectiveness in immunocompromised adults was assumed to be lower based on data from a single trial of children with HIV and without HIV, respectively [16]. We note that PCV13 has demonstrated immunogenicity and safety in several patient populations considered to be at high risk due to the presence of immunocompromising conditions or treatments [35–40].

Another area of parameter uncertainty concerns rates of IPD and pneumococcal CAP, not only in the first year of the modelling horizon, but also future levels of disease that may be prevented with vaccination. The percentage of CAP that is attributable to *S*. *pneumoniae* among Belgian adults is largely unknown; the conservative estimates from Blommaert et al. were applied [10]. Rates of disease were adjusted for projected herd effects from switch vaccination to PCV10 in children. However, the precise nature of herd effects, including corresponding levels and rates of change over time (and the serotypes affected), is largely unknown at this time (especially for pneumococcal CAP). Approach considered in Blommaert et al. [10] with PCV10 vaccination in children was applied in our analysis. Moreover, sentinel laboratories—from which IPD rates were calculated—were assumed to be geographically representative across Belgium and to have reported all diagnosed cases of *S*. *pneumoniae*, the veracity of which is unknown. Because the purpose of this study was to compare the risks and costs of IPD and pneumococcal CAP over remaining years of life for a given cohort of Belgian adults assumed to receive PCV13 versus no vaccine, persons were precluded from aging into the model population.

We also note that PCV13 coverage levels were based on the assumption that the vaccine will be reimbursed, and that its use will be comparable to other vaccines that are currently reimbursed in Belgium (e.g., influenza, which can be administered at the same time as PCV13). Finally, our model, like all cohort models, simplifies reality to some extent. Our model does not, for example, take into account serotype replacement over time; to date, replacement by non-PCV serotypes appears to be limited in the Belgian adult population, however, and thus its consideration in the model would be expected to have a limited impact on study findings based upon the data and time period that were considered [41]. Long term consequences due to pneumococcal infections were also not considered.

## Conclusion

The results of the pharmacoeconomic assessment described herein suggest that, under conservative assumptions concerning disease burden (incidence and mortality), and reasonable assumptions on vaccine effectiveness, and cost of pneumococcal infection, implementation of a strategy targeting Belgian moderate-risk and high-risk adults aged 65–84 years for vaccination with PCV13 would reduce the numbers of cases of pneumococcal disease and pneumococcal-related deaths and would be—on an overall basis—cost effective from the healthcare public system perspective.

## Supporting information

S1 File Cost-effectiveness of PCV13 in moderate/high-risk Belgian adults aged 65–84 years. (DOC)

## References

[1] Verhaegen J, Flamaing J, De Backer W, Delaere B, Van Herck K, Surmont F, et al Epidemiology and outcome of invasive pneumococcal disease among adults in Belgium, 2009–2011. Euro Surveill. 2014; 19(31): 14–22. 10.2807/1560-7917.es2014.19.31.20869 25138972

[2] Wroe PC, Finkelstein JA, Ray GT, Linder JA, Johnson KM, Rifas-Shiman S, et al Aging Population and Future Burden of Pneumococcal Pneumonia in the United States. J Infect Dis. 2012; 205(10): 1589–1592. doi: 10.1093/infdis/jis240 22448012 10.1093/infdis/jis240

[3] Huang SS, Johnson KM, Ray GT, Wroe P, Lieu TA, Moore MR, et al Healthcare utilization and cost of pneumococcal disease in the United States. Vaccine. 2011; 29(18): 3398–3412. doi: 10.1016/j.vaccine.2011.02.088 21397721 10.1016/j.vaccine.2011.02.088

[4] Weycker D, Strutton D, Edelsberg J, Sato R, Jackson LA. Clinical and economic burden of pneumococcal disease in older US adults. Vaccine. 2010; 28(31): 4955–4960. doi: 10.1016/j.vaccine.2010.05.030 20576535 10.1016/j.vaccine.2010.05.030

[5] Institute of Public Health, WIV-ISP 2013. Health Interview Survey. Available from: https://his.wiv-isp.be. [Accessed on June 22, 2014]

[6] Bonten MJ, Huijts SM, Bolkenbaas M, Webber C, Patterson S, Gault S, et al Polysaccharide Conjugate Vaccine against Pneumococcal Pneumonia in Adults. N Engl J Med. 2015; 372(12): 1114–1125. doi: 10.1056/NEJMoa1408544 25785969 10.1056/NEJMoa1408544

[7] Conseil Supérieur de le Santé. (2014). CSS9210. Vaccination antipneumococcique des adultes. Available from: http://www.health.belgium.be/fr/vaccination-antipneumococcique-adultes-2014-css-9210. [Accessed on July 27, 2015]

[8] Suaya JA, Jiang Q, Scott DA, Gruber WC, Webber C, Schmoele-Thoma B, Hall-Murray CK, Jodar L, Isturiz RE. Post hoc analysis of the efficacy of the 13-valent pneumococcal conjugate vaccine against vaccine-type community-acquired pneumonia in at-risk older adults. Vaccine. 2018; 36(11): 1477–1483. doi: 10.1016/j.vaccine.2018.01.049 29429807 10.1016/j.vaccine.2018.01.049

[9] Blommaert A, Bilcke J, Willem L, Verhaegen J, Goossens H, Beutels P. The cost-effectiveness of pneumococcal vaccination in healthy adults over 50: an exploration of influential factors for Belgium. Vaccine. 2016; 34(18): 2106–2112. doi: 10.1016/j.vaccine.2016.03.003 26988257 10.1016/j.vaccine.2016.03.003

[10] Blommaert A, Hanquet G, Willem L, Theeten H, Thiry N, Bilcke J, et al Use of pneumococcal vaccines in the elderly: an economic evaluation. Health Technology Assessment (HTA) Brussels: Belgian Health Care Knowledge Centre (KCE) 2016 KCE report 274. D/2016/10.273/79. Available from: https://kce.fgov.be/sites/default/files/page_documents/KCE_274_Pneumococcal_Report.pdf. [Accessed on January 15, 2017]

[11] Weycker D, Sato R, Strutton D, Edelsberg J, Atwood M, Jackson LA. Public health and economic impact of 13-valent pneumococcal conjugate vaccine in US adults aged >50 years. Vaccine. 2012; 30(36): 5437–5444. doi: 10.1016/j.vaccine.2012.05.076 22728289 10.1016/j.vaccine.2012.05.076

[12] Scientific Institute of Public Health, HISIA: Belgian health interview survey–Interactive analysis. WIV-ISP 2013. Institut Santé Publique. Enquête de Santé. Available from: https://hisia.wiv-isp.be/SitePages/Home.aspx. [Accessed on July 15, 2015]

[13] van Hoek AJ, Andrews N, Waight PA, et al The effect of underlying clinical conditions on the risk of developing invasive pneumococcal disease in England. J Infect 2012;65(1):17–24. doi: 10.1016/j.jinf.2012.02.017 22394683 10.1016/j.jinf.2012.02.017

[14] Truyers C, Goderis G, Dewitte H, et al The Intego database: background, methods and basic results of a Flemish general practice-based continuous morbidity registration project. BMC Med Inform Decis Mak 2014;14:48 doi: 10.1186/1472-6947-14-48 24906941 10.1186/1472-6947-14-48 PMC4067630

[15] van Werkhoven CH, Huijts SM, Bolkenbaas M, Grobbee DE, Bonten MJ. The Impact of Age on the Efficacy of 13-valent Pneumococcal Conjugate Vaccine in Elderly. Clin Infect Dis. 2015 12 15;61(12):1835–8. doi: 10.1093/cid/civ686 26265498 10.1093/cid/civ686

[16] Klugman KP, Madhi SA, Huebner RE, Kohberger R, Mbelle N, Pierce N; Vaccine Trialists Group. A trial of a 9-valent pneumococcal conjugate vaccine in children with and those without HIV infection. N Engl J Med. 2003; 349(14): 1341–1348. doi: 10.1056/NEJMoa035060 14523142 10.1056/NEJMoa035060

[17] Suaya J A, Jiang Q, Bonten M et al., Post-hoc analysis of the 13-valent polysaccharide conjugate vaccine efficacy against vaccine-serotype pneumococcal community acquired pneumonia in at-risk older adults, ISPPD poster presentation in 2016, Glasgow, UK. Submitted to PLoSOne.

[18] Mangen MJ, Rozenbaum MH, Huijts SM, van Werkhoven CH, Postma DF, Atwood M, et al Cost-effectiveness of adult pneumococcal conjugate vaccination in the Netherlands. Eur Respir J. 2015; 46(5): 1407–1416. doi: 10.1183/13993003.00325-2015 26160871 10.1183/13993003.00325-2015 PMC4750466

[19] Beutels P, Van Damme P., Oosterhuis-Kafeja F. Effects and costs of pneumococcal conjugate vaccination of Belgian children. Health Technology Assessment (HTA) Brussels: Belgian Health Care Knowledge Centre (KCE): 2006. KCE reports 33C (D/2006/10.273/53)

[20] Beutels P, Blommaert A, Hanquet G, Bilcke J, Thiry N, Sabbe M, et al Cost-effectiveness of 10- and 13-valent pneumococcal conjugate vaccines in childhood. Health Technology Assessment (HTA) Brussels: Belgian Health Care Knowledge Centre (KCE) 2011 Report 155 C. D/2011/10.273/21. Available from: https://kce.fgov.be/sites/default/files/page_documents/kce_155c_pneumococcal_vaccines.pdf. [Accessed on March 15, 2015]

[21] Weycker D, Farkouh R, Strutton D, et al Rates and costs of invasive pneumococcal disease and pneumonia in persons with underlying medical conditions. BMC Health Serv Res. 2016; 16:182 doi: 10.1186/s12913-016-1432-4 27177430 10.1186/s12913-016-1432-4 PMC4867996

[22] CBIP BCFI 2015. Répertoire Commenté des médicaments. Available at http://www.cbip.be/fr/start. [Accessed March 2015]

[23] Institut National d'assurance Maladie-Invalidité. Soins de santé: Prix et honoraires. Available at: www.inami.fgov.be. [Accessed March 2015]

[24] Stoecker C, Kim L, Gierke R, Pilishvili T. Incremental cost-effectiveness of 13-valent pneumococcal conjugate vaccine for adults age 50 years and older in the United States. J Gen Intern Med. 2016; 31(8): 901–908. doi: 10.1007/s11606-016-3651-0 26976292 10.1007/s11606-016-3651-0 PMC4945555

[25] Rodriquez Gonzalez-Moro JM, Menendez R, Campins M, Lwoff N, Oyaguez I, Echave M, et al Cost-effectiveness of the 13-valent pneumococcal conjugate vaccination program in chronic obstructive pulmonary disease patients aged 50+ years in Spain. Clin Drug Investig. 2016; 36(1): 41–53. doi: 10.1007/s40261-015-0345-z 26547199 10.1007/s40261-015-0345-z PMC4706838

[26] Hoshi SL, Kondo M, Okubo I. Economic evaluation of immunisation programme of 23-valent pneumococcal polysaccharide vaccine and the inclusion of 13-valent pneumococcal conjugate vaccine in the list for single-dose subsidy to the elderly in Japan. PloS One. 2015; 10(10): e0139140 doi: 10.1371/journal.pone.0139140 26444287 10.1371/journal.pone.0139140 PMC4596483

[27] van Hoek AJ, Miller E. Cost-effectiveness of vaccinating immunocompetent ≥65 year olds with the 13-valent pneumococcal conjugate vaccine in England. PloS One. 2016; 11(2): e0149540 doi: 10.1371/journal.pone.0149540 26914907 10.1371/journal.pone.0149540 PMC4767406

[28] Chen J, O’Brien MA, Yang HK, Grabenstein JD, Dasbach EJ. Cost-effectiveness of pneumococcal vaccines for adults in the United States. Adv Ther. 2014; 31(4): 392–409. doi: 10.1007/s12325-014-0115-y 24718851 10.1007/s12325-014-0115-y PMC4003344

[29] Jiang Y, Gauthier A, Keeping S, Carroll S. Cost-effectiveness of vaccinating the elderly and at-risk adults with the 23-valent pneumococcal polysaccharide vaccine or 13-valent pneumococcal conjugate vaccine in the UK. Expert Rev Pharmacoecon Outcomes Res. 2014; 14(6): 913–927. doi: 10.1586/14737167.2014.950232 25189087 10.1586/14737167.2014.950232

[30] Cho BH, Stoecker C, Link-Gelles R, Moore MR. Cost-effectiveness of administering 13-valent pneumococcal conjugate vaccine in addition to 23-valent pneumococcal polysaccharide vaccine to adults with immunocompromising conditions. Vaccine. 2013; 31(50): 6011–6021. doi: 10.1016/j.vaccine.2013.10.024 24148572 10.1016/j.vaccine.2013.10.024

[31] Smith KJ, Nowalk MP, Raymund M, Zimmerman RK. Cost-effectiveness of pneumococcal conjugate vaccination in immunocompromised adults. Vaccine. 2013; 31(37): 3950–3956. doi: 10.1016/j.vaccine.2013.06.037 23806240 10.1016/j.vaccine.2013.06.037 PMC3742552

[32] Zimmerman RK, Lauderdale DS, Tan SM, Wagener DK. Prevalence of high-risk indications for influenza vaccine varies by age, race, and income. Vaccine. 2010; 28(39): 6470–7. doi: 10.1016/j.vaccine.2010.07.037 20674882 10.1016/j.vaccine.2010.07.037 PMC2939262

[33] Smith KJ, Zimmerman RK, Lin CJ, Nowalk MP, Ko FS, McEllistrem MC, et al Alternative strategies for adult pneumococcal polysaccharide vaccination: a cost-effectiveness analysis. Vaccine. 2008; 26(11): 1420–1431. doi: 10.1016/j.vaccine.2008.01.007 18272262 10.1016/j.vaccine.2008.01.007

[34] Hak E, Grobbee DE, Sanders EA, Verheij TJ, Bolkenbaas M, Huijts SM, et al Rationale and design of CAPITA: a RCT of 13-valent conjugated pneumococcal vaccine efficacy among older adults. Neth J Med. 2008; 66(9): 378–383. 18990781

[35] Bhorat AE, Madhi SA, Laudat F, Sundaraiyer V, Gurtman A, Jansen KU, Scott DA, Emini EA, Gruber WC, Schmoele-Thoma B. Immunogenicity and safety of the 13-valent pneumococcal conjugate vaccine in HIV-infected individuals naive to pneumococcal vaccination. AIDS. 2015 7 17;29(11):1345–54 doi: 10.1097/QAD.0000000000000689 25888646 10.1097/QAD.0000000000000689 PMC4521829

[36] Cordonnier C, Ljungman P, Juergens C, Maertens J, Selleslag D, et al; 3003 Study Group. Immunogenicity, safety, and tolerability of 13-valent pneumococcal conjugate vaccine followed by 23-valent pneumococcal polysaccharide vaccine in recipients of allogeneic hematopoietic stem cell transplant aged ≥2 years: an open-label study. Clin Infect Dis. 2015 8 1;61(3):313–23 doi: 10.1093/cid/civ287 25870329 10.1093/cid/civ287 PMC4503811

[37] De Montalembert M, Abboud MR, Fiquet A, Inati A, Lebensburger JD, Kaddah N, et al 13-valent pneumococcal conjugate vaccine (PCV13) is immunogenic and safe in children 6–17 years of age with sickle cell disease previously vaccinated with 23-valent pneumococcal polysaccharide vaccine (PPSV23): Results of a phase 3 study. Pediatr Blood Cancer. 2015 8;62(8):1427–36. doi: 10.1002/pbc.25502 25810327 10.1002/pbc.25502

[38] Hung TY, Kotecha RS, Blyth CC, Steed SK, Thornton RB, Ryan AL, Cole CH, Richmond PC. Immunogenicity and safety of single-dose, 13-valent pneumococcal conjugate vaccine in pediatric and adolescent oncology patients. Cancer. 2017 7 11 doi: 10.1002/cncr.30764 [Epub ahead of print] 28696530 10.1002/cncr.30764

[39] Jallow S, Madhi SA, Madimabe R, Sipambo N, Violari A, Kala U, Petersen K, Naidoo S, Verwey C, Moore DP, Nunes MC. Immunogenicity of 13-valent pneumococcal conjugate vaccine among children with underlying medical conditions. Vaccine. 2017 8 3;35(34):4321–4329. doi: 10.1016/j.vaccine.2017.06.081 28688781 10.1016/j.vaccine.2017.06.081

[40] Lombardi F, Belmonti S, Fabbiani M, Morandi M, Rossetti B, Tordini G, Cauda R, De Luca A, Di Giambenedetto S, Montagnani F. Immunogenicity and Safety of the 13-Valent Pneumococcal Conjugate Vaccine versus the 23-Valent Polysaccharide Vaccine in Unvaccinated HIV-Infected Adults: A Pilot, Prospective Controlled Study. PLoS One. 2016 6 3;11(6):e0156523 doi: 10.1371/journal.pone.0156523 eCollection 2016. 27258647 10.1371/journal.pone.0156523 PMC4892598

[41] van Werkhoven CH, Huijts SM, Bolkenbaas M, Webber C, Hollingsworth R, Patterson S, et al. Herd effects of infant immunisation with pneumococcal conjugate vaccines. A post-hoc analysis of the CAP-pilot study and Community-Acquired Pneumonia immunisation Trial in Adults (CAPiTA), poster presented at 25th European Congress of Clinical Microbiology and Infectious Diseases (ECCMID), 25–28 April 2015, Copenhagen, Denmark.

