# Supplementary material for: Cost-effectiveness of PCV13 vaccination in Belgian adults aged 65-84 years at elevated risk of pneumococcal infection
Source: PLoS One. 2018 Jul 6;13(7):e0199427. doi: 10.1371/journal.pone.0199427 (PMC6034794; doi:10.1371/journal.pone.0199427)

**S1 ONLINE SUPPLEMENT FOR Cost-Effectiveness of PCV13 in**

**Moderate/High-Risk Belgian Adults Aged 65-84 Years:**

**Details on Estimation of Model Parameter Values**

1. **MODEL POPULATION**

Population data comes from the SPF economy website (http://statbel.fgov.be/fr/modules/publications/statistiques/population/population_totale_et_belge_au_1_janvier_2001-2007_.jsp, access on February 7, 2017). We have considered the year 2015 for the analysis. Based on these data, the number of Belgian population aged 65-84 y. is as follows:

***Table 1: Belgian adult*** population in 2015

| **Age groups** | **N** |
| --- | --- |
| 65-74 y | 1,051,936 |
| 75-84 y | 711,869 |

To determine the % of this population that is at risk of pneumococcal infections, we refer to data from INTEGO database, making distinction between the high-risk group (= immunocompromised patients) and the moderate-risk group (= patients with co-morbidities):

| ***Table 2. Proportion of patients at risk in 2013 (Total population in Intego, n = 95,508)*** |
| --- |

|  |  | | | Total population | | **65 – 74 y.** | **75 – 84 y.** | | **≥85 y.** |
| --- | --- | --- | --- | --- | --- | --- | --- | --- | --- |
|  |  |  |  |  |  | (n= 10256) | (n=7960) | | (n= 4072) |
| **High risk group, n** | | | | **2287** | | **435** | **342** | | **95** |
|  | Aids, n (%) | | | 91 (4.0) | | 2 (0,09) | 0 | | 0 |
|  | Lymphoma, n (%) | | | 126 (5.5) | | 33 (1,44) | 13 (0,57) | | 3 (0,13) |
|  | Leukemia, n (%) | | | 147 (6.4) | | 23 (1,01) | 22 (0,96) | | 6 (0,26) |
|  | Malignant neoplasm blood other, n (%) | | | 55 (2.4) | | 18 (0,79) | 12 (0,52) | | 3 (0,13) |
|  | Immunosuppressant, n (%) | | | 650 (28) | | 123 (5,38) | 84 (3,67) | | 11 (0,48) |
|  | Glucocorticoids, n (%) | | | 1315 (57) | | 255 (11,15) | 232 (10,14) | | 77 (3,37) |
| **Median risk group, n** | | | | **22889** | | **4105** | **4092** | | **2212** |
|  | Chronic bronchitis, n (%) | | | 907 (3.9) | | 184 (0,8) | 165 (0,72) | | 83 (0,36) |
|  | Congenital respiratory anomaly, n (%) | | | 18 (0.08) | | 2 (0,009) | 5 (0,022) | | 0 (0) |
|  | COPD, n (%) | | | 2370 (10) | | 570 (2,5) | 535 (2,3) | | 241 (1,1) |
|  | Asthma, n (%) | | | 9061 (40) | | 1227 (5,37) | 815 (3,56) | | 241 (1,05) |
|  | Congenital cardiovascular anomaly, n (%) | | | 244 (1.1) | | 32 (0,14) | 22 (0,096) | | 7 (0,031) |
|  | Angina pectoris, n (%) | | | 253 (1.1) | | 57 (0,25) | 68 (0,30) | | 24 (0,11) |
|  | Acute myocardial infarction, n (%) | | | 1910 (8.3) | | 399 (1,75) | 386 (1,69) | | 197 (0,86) |
|  | Chronic ischemic heart disease, n (%) | | | 1236 (5.4) | | 319 (1,4) | 376 (1,64) | | 174 (0,76) |
|  | Heart failure, n (%) | | | 957 (4.2) | | 143 (0,63) | 322 (1,41) | | 366 (1,6) |
|  | Atrial fibrillation, n (%) | | | 2147 (9.4) | | 489 (2,14) | 745 (3,26) | | 536 (2,34) |
|  | Pulmonary heart disease, n (%) | | | 173 (0.76) | | 36 (0,16) | 72 (0,32) | | 47 (0,21) |
|  | Heart valve disease, n (%) | | | 1446 (6.3) | | 298 (1,3) | 422 (1,85) | | 256 (1,12) |
|  | TIA, n (%) | | | 990 (4.3) | | 218 (0,95) | 310 (1,35) | | 302 (1,32) |
|  | CVA, n (%) | | | 1778 (7.8) | | 352 (1,54) | 436 (1,9) | | 347 (1,51) |
|  | Peripheral arterial disease, n (%) | | | 1895 (8.3) | | 418 (1,83) | 539 (2,35) | | 274 (1,2) |
|  | Chronic liver disease, n (%) | | | 1705 (7.4) | | 358 (1,56) | 292 (1,27) | | 98 (0,43) |
|  | Chronic kidney disease, n (%) | | | 5421 (24) | | 1211 (5,29) | 1951 (8,53) | | 1229 (5,37) |
|  | Chronic alcohol abuse, n (%) | | | 1652 (7.2) | | 263 (1,15) | 107 (0,47) | | 29 (0,12) |
|  |  |  |  |  |  |  |  |  |  |
| **Low risk patients, n** | | | | **70332** | | **5716** | **3526** | | **1765** |

COPD: chronic obstructive pulmonary disease; TIA: transient ischemic attack; CVA: cerebrovascular accident.

The definition of these risk groups is consistent with the definition of these in the recommendations by the Superior Health Council.

Based on these data, the proportion of Belgian population at risk of pneumococcal infections has been defined as follows:

***Table 3: Proportion of patients at moderate and high risk of pneumococcal disease in 2013***

| **Age groups** | **65-74 y.** | **75-84 y.** | **85+y.** |
| --- | --- | --- | --- |
| **High risk (immunocompromised)** | 4.2% | 4.3% | 2.3% |
| **Moderate risk**  **(one or more chronic diseases)** | 40.0% | 51.4% | 54.3% |

*Source: INTEGO database 2013*

These percentages are in line with the percentage as observed in the Netherlands (Mangen 2015; Stirbu-Wagner 2010) and in UK (van Hoek 2012) and are applied for following years. In Weycker et al. (2016), the percentage of high risk patients was larger due to the high number of patients using immunosuppressants, reaching 12% based on the same definition as hereabove.

These percentages are also in line with the results from the Belgium National Health Survey 2013 (namely, based on the proportion of the subjects with a chronic condition or handicap, considered as the definition for the patients with moderate risk)

1. **DISEASE INCIDENCE**
   1. **Invasive Pneumococcal Disease**

The national incidence of IPD (bacteraemia and meningitis) per age group was derived from the surveillance made by the Belgian National Reference Centre and corrected to the whole Belgian population (KCE report, 2016 – Blommaert et al., 2016b). As mentioned by the authors of the KCE report, this estimation is expected to be conservative and will consequently be subject to sensitivity analysis.

***Table 4: Estimation of the incidence of IPD (excluding meningitis) per 100.000 Belgian inhabitants in 15 (KCE report- Blommaert et al., 2016b)***

| **Age groups** | **Incidence estimation of IPD (excluding meningitis) per 100.000 inhabitants** |
| --- | --- |
| 65-74 y | 24.3 |
| 75-84 y | 35.2 |
| 85 y+ | 76.6 |

*Source: KCE report, 2016 (incidence invasive pneumonia and other IPD)*

***Table 5****:* ***Estimation of the national incidence meningitis per 100.000 Belgian inhabitants in 2015***

| **Age groups** | **Incidence meningitis per 100.000 inhabitants** |
| --- | --- |
| 65-74 y | 1.1 |
| 75-84 y | 1.6 |
| 85 y+ | 3.6 |

*Source: KCE report, 2016*

The incidence differs by age and by risk profile. We do not have IPD incidence data by risk group. We have extrapolated the difference in incidence by risk profile based on a UK publication (van Hoek 2012). Based on the results of this publication, we have calculated relative risk ratio:

***Table 6: Relative risk of incidence rate***

| **Age Groups** | **Relative Risk Ratios** | | |
| --- | --- | --- | --- |
|  | **Low** | **Moderate** | **High** |
|  |  |  |  |
| 65-74 y | 1.0 | 3.5 | 7.7 |
| 75-84 y | 1.0 | 3.5 | 7.7 |
| 85 y+ | 1.0 | 3.5 | 7.7 |

*Source : van Hoek 2012*

Based on these relative risk ratios and the proportion of low, moderate and high risk in the Belgian population within these age groups, we have estimated the following incidence rate of IPD per age group and risk profile (table 7). These are very conservative compared with the RR ratios used in the scenario analysis of Blommaert et al. (2016b), namely 2 for the moderate risk group vs. the all risk groups.

***Table 7: Extrapolation of age-specific IPD rates (excluding meningitis) to age and risk-specific rates***

|  | **IPD (excluding meningitis) incidence per 100,000 inhabitants** | | | | |
| --- | --- | --- | --- | --- | --- |
| **Age Groups** | **All Risk Groups** | **Low Risk** | | **Moderate Risk** | **High Risk** |
| 65-74 y | 24.30 | 10.58 | | 37.39 | 81.96 |
| 75-84 y | 35.20 | 13.58 | | 47.98 | 105.18 |
| 85 y+ | 76.60 | 30.26 | | 106.95 | 234.45 |
|  |  |  | |  |  |
|  | **Meningitis Incidence per 100,000 inhabitants** | | | | |
| **Age Groups** | **All Risk Groups** | **Low Risk** | **Moderate Risk** | | **High Risk** |
| 65-74 y | 1.10 | 0.48 | 1.69 | | 3.71 |
| 75-84 y | 1.60 | 0.62 | 2.18 | | 4.78 |
| 85 y+ | 3.60 | 1.42 | 5.03 | | 11.02 |

*Source: KCE report (Blommaert et al., 2016b) , van Hoeck (2012)*

- 1. **Pneumococcal non-invasive Pneumonia -- Hospitalized**

We have considered the incidence of the hospitalized pneumococcal non-invasive CAP as estimated in the KCE report (Blommaert et al., 2016b). As mentioned by the authors, this estimation, based on the estimation of the IPD incidence is conservative, and will consequently as well be subject to sensitivity analysis.

***Table 8: Estimation national incidence hospitalized pneumococcal CAP per 100,000 Belgian inhabitants, per age range (2015)***

| **Age groups** | **N/100 000 inhabitants** |
| --- | --- |
| 65-74 y | 105.9 |
| 75-84 y. | 152.7 |
| 85 y+ | 332.8 |

Because only age-specific (and not age- and risk-specific) rates of hospitalized CAP were available from the source material, age-specific rate ratios for moderate/high-risk vs. low-risk were derived based on outpatient CAP data (Intego database).

| **Age Groups** | **Relative Risk Ratios** | | |
| --- | --- | --- | --- |
|  | **Low** | **Moderate** | **High** |
| 65-74 y | 1.0 | 2.01 | 6.83 |
| 75-84 y | 1.0 | 2.64 | 8.49 |
| 85 y+ | 1.0 | 2.70 | 8.57 |

*Source: INTEGO*

These rate ratios were then used to allocate age-specific rates of hospitalized CAP across risk groups. These are very conservative compared with the RR ratios used in the scenario analysis of Blommaert et al. (2016b), namely 2 for the moderate risk group vs. the all risk groups.

***Table 9: Estimation national incidence hospitalized pneumococcal*** CAP per 100,000 Belgian inhabitants, per age and risk profile:

|  | **Incidence per 100,000 inhabitants** | | | |
| --- | --- | --- | --- | --- |
| **Age Groups** | **All Risk Groups** | **Low Risk** | **Moderate Risk** | **High Risk** |
| 65-74 y. | 105.9 | 64.30 | 128.92 | 439.34 |
| 75-84 y. | 152.7 | 70.60 | 186.09 | 599.44 |
| 85 y+ | 332.8 | 202.06 | 545.70 | 1,732.66 |
| *Source: KCE report (Blommaert A. et al, 2016b), extrapolation per risk group based on outpatient split (INTEGO)* | | | | |

- 1. **Pneumococcal Pneumonia -- Outpatient Care**

For the outpatient pneumonia per age and group profile, we have used data from INTEGO database. The last available data were from year 2013. We have selected the code R81 ‘Pneumonia’ from the ICPC classification. Data from this GP network were then corrected for extrapolation to all Belgian population, in line with KCE report (Blommaert A. et al., 2016b).

Based on 2 publications from Spain and Denmark, KCE authors considered that 10.5% of all cause-CAP as derived from the INTEGO database are from pneumococcal origin. Based on other papers and literature review (Torres A. et al., 2014) as well as the Blommaert’s paper (2016a), this 10.5% is considered by the clinicians as an underestimation, based also on the fact that the pneumococcal infection is underdiagnosed at GP level. We keep a conservative approach by considering a 15% of all cause outpatient CAP being from pneumococcal origin, which remains far lower than the % referred in Blommaert et al. (2016a).

The incidence of outpatient pneumococcal pneumonia as derived from the INTEGO database and considering 15% of pneumococcus origin was as followed, per age and risk group:

|  | **Incidence per 100,000 inhabitants** | | | |
| --- | --- | --- | --- | --- |
| **Age Groups** | **All Risk Groups** | **Low Risk** | **Moderate Risk** | **High Risk** |
| 65-74 y. | 73.48 | 44.61 | 89.45 | 304.82 |
| 75-84 y. | 110.21 | 50.96 | 134.31 | 432.65 |
| 85 y+ | 164.43 | 78.39 | 206.61 | 665.56 |

*Source: KCE report (Blommaert A. et al, 2016b), corrected with 15% of pneumococcal infection with the all-cause CAP*

|  |
| --- |

1. **INDIRECT EFFECTS**

As of 2016, the childhood vaccination program switched from PCV13 to PCV10. The indirect effect caused by the lower serotype coverage and limited indirect effect observed with PCV10 is uncertain. Some insights are given based on serotypes incidence in the Netherlands and in Finland. In Finland Serotypes 19A, not included in the PCV10 vaccine used in the children vaccination programme, is strongly increasing in the older adult population (National Institute of Health and Welfare Finland, 2016). It can be expected that serotype 19A, as well as 3 and 6A will re-emerge in Belgium. The Netherlands also observed an increase in the non PCV10 IPD since the switch from the PVC7 to PCV10.

Based on the recommendations from the KCE authors as well as from clinicians with experience in the Netherlands (Mangen et al., 2015), we considered a similar approach as the quick relapse scenario described in the KCE report (Blommaert et al., 2016b). This scenario considers indirect effect consequently to the switch of children vaccination to PCV10 and a progressive increase in incidence after residual indirect effect during 2 years.

- 2 first years after switch to PCV10 vaccination in children: - 16% annually (residual indirect effect from previous PCV13 vaccination in children)
- As from year 3 up to year 7 after: 7.2% annual increase to reach baseline incidence rate of year 2015 at year 7
- As from year 7, stabilization

1. **MORTALITY**
   1. **General Population**

Data on mortality rates for the general population were obtained from the SPF economy website (SPF 2015).

***Table 10: Mortality rate in general population***

| **Age groups** | **Mortality rate in general population (2015)** |
| --- | --- |
| 65 – 74 y. | 1.57% |
| 75-84 y. | 4.49% |
| 85 y+ | 15.20% |

*Source : http://statbel.fgov.be/fr/statistiques/chiffres/population/deces_mort_esp_vie/tables/*

Based on UK data (van Hoek 2012), we have extrapolated this mortality rate in general population per risk group. These are very conservative compared with the RR ratios used in the scenario analysis of Blommaert et al. (2016b), namely 1.5 for the moderate risk group vs. the all risk groups.

***Table 11: RR ratios***

|  |  | **Relative Risk Ratios** | | | | | | | |
| --- | --- | --- | --- | --- | --- | --- | --- | --- | --- |
| **Age Groups** |  | **Low** | | **Moderate** | | | **High** | | |
| 65-74 y. |  | 1.00 | | 1.20 | | | 1.20 | | |
| 75-84 y. |  | 1.00 | | 1.20 | | | 1.20 | | |
| 85 y+ |  | 1.00 | | 1.20 | | | 1.20 | | |
| *Source: van Hoek 2012* | | | | | | | | | |
|  |  | |  | | |  | | |  |
| ***Table 12: Extrapolation of age-specific general mortality rates (per 100) to age and risk-specific rates*** | | | | | | | | | |
|  | **Mortality rate per 100** | | | | | | | | |
| **Age Groups** | **All Risk Groups** | | **Low Risk** | | **Moderate Risk** | | | **High Risk** | |
| 65-74 y. | 1.57 | | 1.44 | | 1.73 | | | 1.73 | |
| 75-84 y. | 4.49 | | 4.04 | | 4.85 | | | 4.85 | |
| 85 y+ | 15.20 | | 13.65 | | 16.39 | | | 16.39 | |

- 1. **Case-Fatality Rates -- Invasive Pneumococcal Disease**

Mortality data from the KCE report (Blommaert et al., 2016b) were used to derive the mortality rates for IPD (meningitis, pneumococcal invasive pneumonia and septicaemia). These case-fatality rates were defined in the report as fatalities rates consequently to hospitalization. Compared to other KCE report and Blommaert et al‘s publication (2016a), these case-fatality rates are far lower. We consequently propose to use the higher rates based on Blommaert 2016a in the sensitivity analysis. The authors of the recent KCE report mention also that their approach may underestimate the total mortality rate, which confirms the need for sensitivity analysis.

- - 1. **Base case (Blommaert et al., 2016b)**

***Table 13: Case-fatality rates in IPD***

| **Age groups** | **meningitis** | **other IPD (weighted pneumonia and septicaemia)** |
| --- | --- | --- |
| 65-74 y. | 11.60% | 12.66% |
| 75-84 y. | 22.20% | 16.25% |
| 85 y+ | 50.00% | 22.40% |

|  | Findings from the study by van Hoek and colleagues (J Infection, 2012) were used to derive risk-specific case-fatality rates for IPD (as well as pneumonia) among elderly persons in the model population. Specifically, we assumed that case-fatality from IPD (and pneumonia) was 20% higher among moderate-risk and high-risk elderly persons (vs. low-risk elderly persons), based on the odds ratio for IPD case-fatality (1.2 [95%CI: 1.1-1.3]) among persons aged ≥65 years with (vs. without) ≥1 risk factor—including moderate-risk and high-risk conditions—reported in Table 4 of the van Hoek study.  A UK estimate was employed for IPD as Belgian-specific data were not available, and an IPD estimate was employed for pneumonia for reasons of consistency and because it was conservative relative to other available (non-Belgian) data. Finally, an overall estimate was used for moderate-risk elderly persons and high-risk elderly persons as condition-specific data from van Hoek were not robust.  ***Table 14: Extrapolation of age-specific IPD other than meningitis case-fatality rates (per 100) to age and risk-specific rates*** | | | | | | | | |
| --- | --- | --- | --- | --- | --- | --- | --- | --- | --- |
|  | | **Mortality rate per 100** | | | | | | | |
| **Age Groups** | | **All Risk Groups** | | **Low Risk** | | **Moderate Risk** | | **High Risk** | |
| 65-74 y. | | 12.66% | | 11.63% | | 13.96% | | 13.96% | |
| 75-84 y. | | 16.25% | | 14.62% | | 17.55% | | 17.55% | |
| 85 y+ | | 22.40% | | 20.12% | | 24.15% | | 24.15% | |
|  |  | |  | |  | |  | |  |
|  | ***Table 15: Extrapolation of age-specific meningitis case-fatality rates (per 100) to age and risk-specific rates*** | | | | | | | | |
|  |  | | **Mortality rate per 100** | | | | | | |
|  | **Age Groups** | | **All Risk Groups** | **Low Risk** | | **Moderate Risk** | | **High Risk** | |
|  | 65-74 y. | | 11.60% | 10.66% | | 12.79% | | 12.79% | |
|  | 75-84 y. | | 22.20% | 19.97% | | 23.97% | | 23.97% | |
|  | 85 y+ | | 50.00% | 44.92% | | 53.90% | | 53.90% | |

- - 1. **Scenario analysis (Blommaert et al., 2016a)**

***Case-fatality rates in IPD***

| **Age groups** | **Meningitis** | **bacteraemia** |
| --- | --- | --- |
| 65-74 y. | 17.6% | 15.9% |
| 75-84 y. | 31.3% | 27.1% |
| 85 y+ | 31.3% | 27.1% |

|  | ***Extrapolation of age-specific bacteraemia case-fatality rates (per 100) to age and risk-specific rates*** | | | | | | | | |
| --- | --- | --- | --- | --- | --- | --- | --- | --- | --- |
|  | | **Mortality rate per 100** | | | | | | | |
| **Age Groups** | | **All Risk Groups** | | **Low Risk** | | **Moderate Risk** | | **High Risk** | |
| 65-74 y. | | 15.90 | | 14.61 | | 17.53 | | 17.53 | |
| 75-84 y. | | 27.10 | | 24.38 | | 29.26 | | 29.26 | |
| 85 y+ | | 27.10 | | 24.34 | | 29.21 | | 29.21 | |
|  |  | |  | |  | |  | |  |
|  | ***Extrapolation of age-specific meningitis case-fatality rates (per 100) to age and risk-specific rates*** | | | | | | | | |
|  |  | | **Mortality rate per 100** | | | | | | |
|  | **Age Groups** | | **All Risk Groups** | **Low Risk** | | **Moderate Risk** | | **High Risk** | |
|  | 65-74 y. | | 17.60 | 16.17 | | 19.40 | | 19.40 | |
|  | 75-84 y. | | 31.30 | 28.16 | | 33.80 | | 33.80 | |
|  | 85 y+ | | 31.30 | 28.12 | | 33.74 | | 33.74 | |

- 1. **Case-Fatality Rates -- Non-invasive Pneumonia**

Mortality data from the KCE report (Blommaert et al., 2016b) were also used for non-invasive pneumonia. These case-fatality rates were derived by applying a hazard ratio on IPD case-death rates. Compared to another Blommaert et al ‘s publication (2013), these case-fatality rates are also far lower. We consequently propose to use them in the sensitivity analysis. The authors of the recent KCE report mention also that their approach may underestimate the total mortality rate, which confirms the need for sensitivity analysis.

- - 1. **Base case (Blommaert et al., 2016b)**

***Table 16: Mortality rate in hospitalized non-invasive CAP***

| **Age groups** | **Hospitalized CAP** |
| --- | --- |
| 65-74 y. | 3.54% |
| 75-84 y. | 5.14% |
| 85 y+ | 8.00% |

| ***Table 17: Extrapolation of age-specific inpatient pneumonia case-fatality rates (per 100) to age and risk-specific rates*** | | | | |
| --- | --- | --- | --- | --- |
|  | **Mortality Rate per 100** | | | |
| **Age Group** | **All Risk Groups** | **Low Risk** | **Moderate Risk** | **High Risk** |
| 65-74 | 3.54 | 3.25 | 3.90 | 3.90 |
| 75-84 | 5.14 | 4.63 | 5.55 | 5.55 |
| 85 y+ | 8.00 | 7.19 | 8.62 | 8.62 |
| **Source:** KCE report 2016, factor risk group van Hoeck | | | | |

- - 1. **Scenario analysis (Blommaert et al., 2013)**

The mortality rates for hospitalized CAP were derived from the ABC Belgian real life study (Blommaert et A. al., 2013).

***Mortality rate in hospitalized non-invasive CAP***

| **Age groups** | **Hospitalized CAP** |
| --- | --- |
| 65-74 y. | 11.8% |
| 75-84 y. | 16.9% |
| 85 y+ | 21.5% |

The age groups in the ABC real life study were somewhat different that the ones used in the model: as such, the ABC study data from 60-69 year old and 70-79 year olds age groups were used to calculate the mortality rate in the 65-74 year old age group. Similar approach was considered for each age group. Based on the same RR as used for mortality in the general population, the mortality rate due to CAP per risk group has been estimated as:

| ***Extrapolation of age-specific inpatient pneumonia case-fatality rates (per 100) to age and risk-specific rates*** | | | | |
| --- | --- | --- | --- | --- |
|  | **Moratlity rate per 100** | | | |
| **Age Groups** | **All Risk Groups** | **Low Risk** | **Moderate Risk** | **High Risk** |
| 65-74 y. | 11.80 | 10.84 | 13.01 | 13.01 |
| 75-84 y. | 16.90 | 15.21 | 18.25 | 18.25 |
| 85+ y. | 21.50 | 19.31 | 23.18 | 23.18 |

Referring the recent KCE report (Blommaert A. et al., 2016), we will also consider case-fatality rates with **outpatient CAP**.

***Table 18: Extrapolation of age-specific outpatient pneumonia case-fatality rates (per 100) to age and risk-specific rates***

|  | **Mortality Rate per 100** | | | |
| --- | --- | --- | --- | --- |
| **Age Groups** | **All Risk Groups** | **Low Risk** | **Moderate Risk** | **High Risk** |
| 65-74 y. | 1.70 | 1.56 | 1.87 | 1.87 |
| 75-84 y. | 1.70 | 1.53 | 1.84 | 1.84 |
| 85y.+ | 1.70 | 1.53 | 1.83 | 1.83 |
| **Source:** KCE study (2016) | | | | |

1. **PATIENT UTILITIES**

Quality-adjusted life years (QALYs) lost due to premature death was calculated by multiplying the lost life-years with corresponding age-specific general population utilities. The utilities from the general population were derived from the Belgium National Health Survey in 2013.

***Table 19***: Utilities in general population

| **Age groups** | **mean** |
| --- | --- |
| 65-74 y. | 0.788 |
| 75-84 y. | 0.709 |
| 85 y+ | 0.623 |

Age-specific utility values were extrapolated across age- and risk-groups using relative risks between risk groups as observed in Sisk et al. (2003):

***Table 20: RR ratios***

|  |  | **Relative Risk Ratios** | | | | | |
| --- | --- | --- | --- | --- | --- | --- | --- |
| **Age Groups** |  | **Low** | | **Moderate** | | **High** | |
| 65-74 y. |  | 1.00 | | 1.00 | | 0.75 | |
| 75-84 y. |  | 1.00 | | 1.00 | | 0.77 | |
| 85 y.+ |  | 1.00 | | 1.00 | | 0.94 | |
| ***Table 21: Extrapolation of age-specific utility to age/risk-specific utility*** | | | | | | |  |
| **Age Groups** | **All Risk Groups** | | **Low Risk** | | **Moderate Risk** | | **High Risk** |
| 65-74 y. | 0.7880 | | 0.7962 | | 0.7962 | | 0.6001 |
| 75-84 y. | 0.7090 | | 0.7162 | | 0.7162 | | 0.5490 |
| 85y.+ | 0.6230 | | 0.6238 | | 0.6238 | | 0.5876 |

Quality of life loss due to non-fatal **inpatient CAP** was derived from a case-control study within CAPiTA. The CHO-CAP study was designed as a piggy-bag study to the CAPiTA-trial. 48,634 participants of CAPiTA, who consented and provided information on health status (EQ-5D), socio-demographic background and comorbidities at the time of vaccination, constituted the source population of CHO-CAP study. To estimate the decrements in EQ-5D (and healthcare cost attributable to the CAP episode), nested matched cohort analyses were conducted within the CHO-CAP study. For each CAP patient, two controls (who did not have CAP at the time the case was identified) were selected. These patients were prospectively followed up to one year. More information can be found in the design article of this study (Mangen 2013). In the CHO-CAP study, the observed utility difference between CAP cases and controls per CAP episode was 0.0709 (Mangen 2015). The same disutility was considered for all risk groups. This disutility is in line with the one used in the KCE report from another source (PNEUMOCOST).

For the disutility in **outpatient CAP**, we have also considered the same reference as in the KCE report (Galante et al., 2011), namely a 0.508 utility (95%CI 0.578-0.691) during 8.5 days for an ambulatory pneumonia. These values are consistent with other sources.

| ***Table 22: Disutility values for outpatient CAP*** | | | | |  |
| --- | --- | --- | --- | --- | --- |
| **Age Groups** | **All risk, over 8.5 days** | **Moderate Risk, over 1 year** | **High Risk, over 1 year** |  |  |
| 65-74 y. | -0.2800 | -0.0066 | -0.0049 |  |  |
| 75-84 y. | -0.2010 | -0.0047 | -0.0036 |  |  |
| 85 y+ | -0.1150 | -0.0027 | -0.0027 |  |  |

*Source: KCE report (Blommaert et al., 2016b) corrected*

For **IPD**, we have applied the same QALY loss as reported in the KCE report (Blommaert A. et al., 2016) and derived from the PNEUMOCOST-survey, **namely 0.1741** over 1 year period. No differences were considered per risk group.

1. **VACCINE EFFECTIVENESS**

**PCV13**

- 1. **Invasive Pneumococcal Disease**

VE-PCV13 against VT-IPD among immunocompetent (i.e., moderate-risk) persons was based on data from the CAPiTA trial (75% for all study subjects; mean age: 73 years) (Bonten 2015, Hak 2008). Protection was assumed to remain constant over the initial 5 years of the modelling horizon, based on the observation that vaccine effectiveness remained stable during the follow-up period (mean, 4 years) in CAPiTA (Mangen 2015).

The impact of age on VE-PCV13 was estimated using a Cox proportional hazard model, with the first episode of confirmed VT pneumococcal CAP as the outcome event (van Werkhoven 2015). This analysis showed a statistically significant decline in VE for increasing age. In this analysis, the vaccine-age interaction was 3.7% per year (not statistically significant) and the age at which the predicted efficacy equalled the VE from the CAPiTA-trial (45.56%) was 76.2 years (Mangen 2015). This slope was extrapolated to the VE against VT-IPD (75%) based on the relative difference in VE as observed in the CAPiTA-trial and the post-hoc analysis. Based on this slope, VE decreased for individuals aged older than 76 years to age 85 years. VE increased for those aged younger than 76 years to those aged 65 years.

VE was considered stable during the first 5 years following vaccination (Bonten 2015), and thereafter assumed to wane annually at a rate of 5% during years 6–10, 10% annually during years 11–15, and no efficacy was assumed from year 16 onwards, consistent with the approach employed in CAPiTA investigators’ publication (Manger 2015). Based on CAPiTA, PCV13 vaccination was assumed not to have serious adverse effects (Bonten 2015).


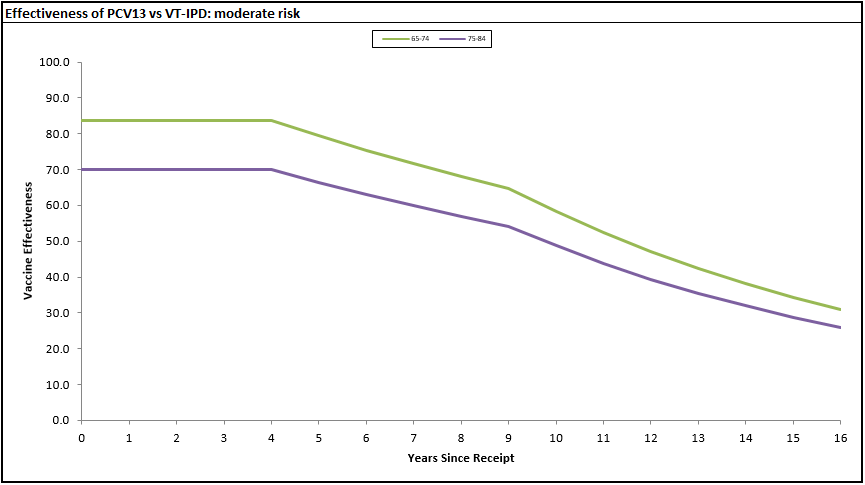


For high-risk adults, VE-PCV13 was assumed to be 22% lower than corresponding values for the immunocompetent group. This assumption was based on a trial of pneumococcal vaccination in children with and without HIV (Klugman 2003). While PCV13 has demonstrated immunogenicity and safety in patients with immunocompromising conditions (Bhorat 2015, Cordonnier 2015, De Montalembert 2015, Lombardi 2016, Hung 2017, Jallow 2017), our assumption is also consistent with the lower efficacy observed in mITT results of the CAPiTA study that included patients who developed immunosuppression during the follow-up period.


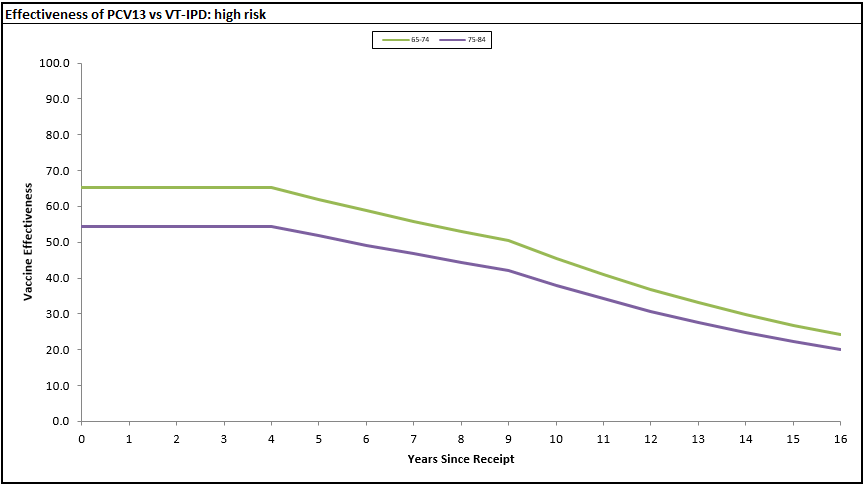


In a scenario analysis, we consider the same approach as in Blommaert et al. (2016b): decline in VE-PCV13 over time (i.e., after the initial 5-year period) was assumed to logistically wane at 75% with 50% of initial vaccine efficacy achieved at year 10.


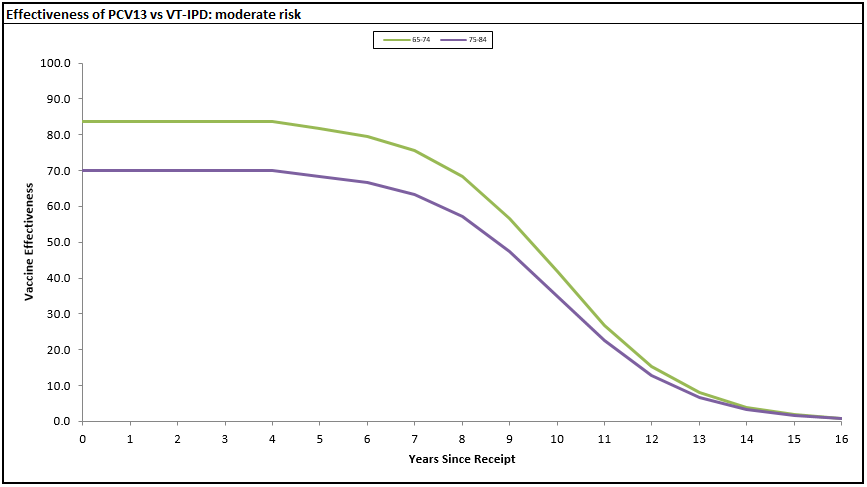


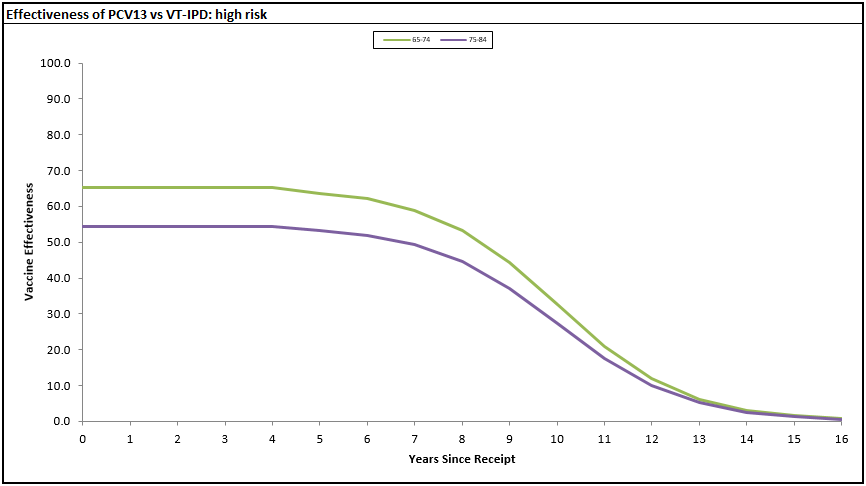


- 1. **Pneumococcal Pneumonia**

VE-PCV13 against vaccine-type pneumonia—irrespective of setting of care—for moderate-risk persons (i.e., those with chronic comorbidities) was derived based on the efficacy of PCV13 against vaccine-type nonbacteremic pneumococcal pneumonia as described in the post-hoc analysis of CAPiTA study (40.28%) (Suaya 2018).

VE-PCV13 against vaccine-type pneumonia was “anchored” to persons aged 73 years (mean age of study subjects in CAPiTA), and change in VE-PCV13 with age (i.e., for persons younger and older than 73 years of age) was equal to 50% of PPV23 values for IPD (Weycker 2012). VE-PCV13 was considered stable during the first 5 years following vaccination (Bonten 2015), and thereafter assumed to wane annually at a rate of 5% during years 6–10, 10% annually during years 11–15, and no efficacy was assumed from year 16 onwards consistent with the approach employed by the CAPiTA investigators’ publication (Mangen 2015). Based on CAPiTA, PCV13 vaccination was assumed not to have serious adverse effects (Bonten 2015).


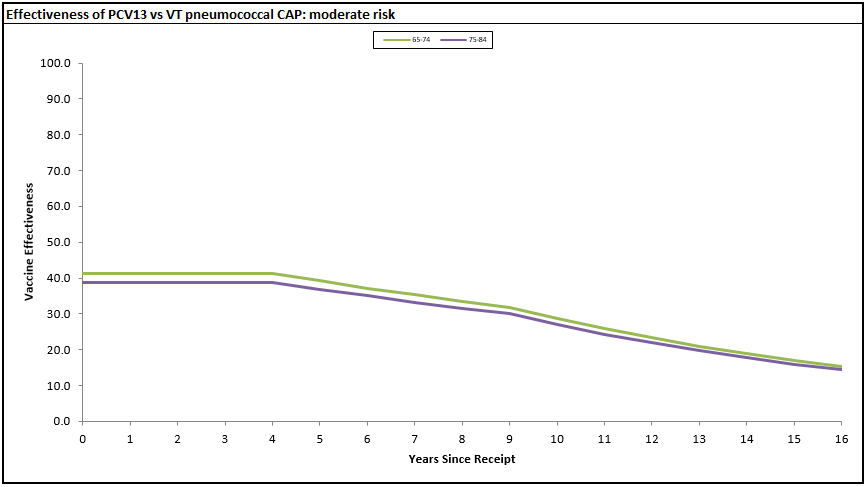


For high-risk adults, VE-PCV13 against pneumococcal CAP was assumed to be 35% lower than the corresponding values for immunocompetent persons based on a trial of pneumococcal vaccination in children with and without HIV (Klugman 2003). This finding also is consistent with the lower efficacy observed in mITT results of the CAPiTA study that included patients who developed immunosuppression during the follow-up period.


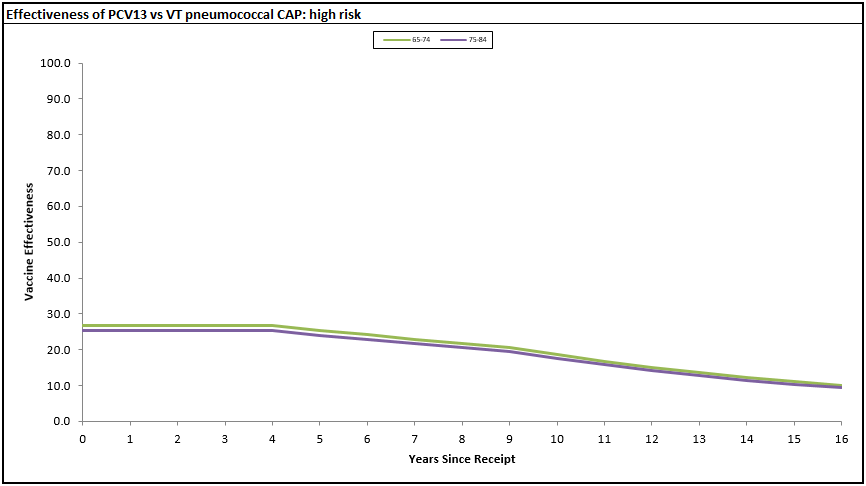


In a scenario analysis, we consider the same approach employed in Blommaert (2016b): decline in VE-PCV13 over time (i.e., after the initial 5-year period) was assumed to logistically wane at 75% with 50% of initial vaccine efficacy achieved at 10 years. VE-PCV13 was assumed to be 0% at year 16 of the modelling horizon.


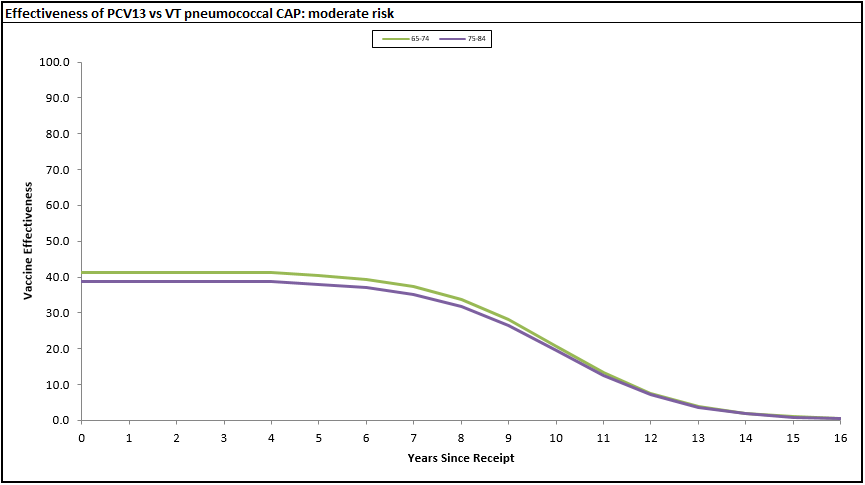


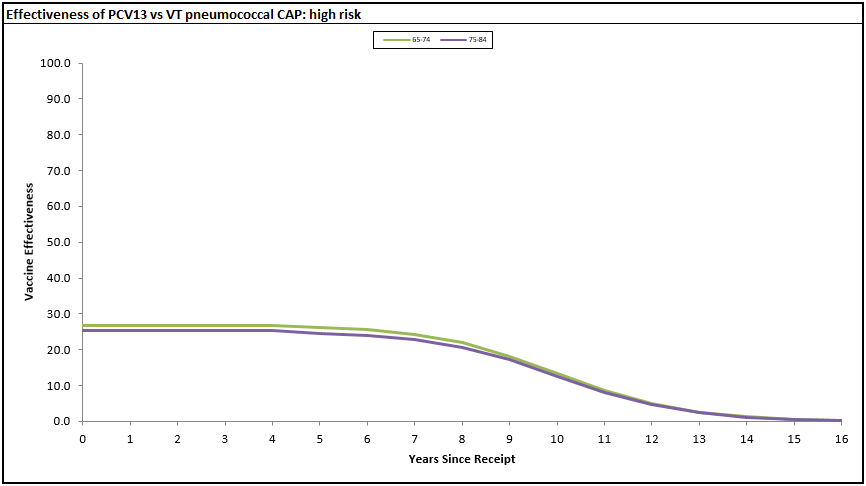


**PPV23**

**Invasive pneumococcal disease**

We referred, as in the KCE report (Blommaert A. et al. 2016b), to the Andrews paper (2012) to define the vaccine type efficacy in risk group, namely:

|  |  |  |  |  | **65-74 y** | **75-84 y** | |
| --- | --- | --- | --- | --- | --- | --- | --- |
| No risk | |  |  |  | 56% | 27% |  |
| Risk group immuno-competent | | | | 21% | | 23% |  |
| Risk group immunocompromised* | | | | -17% | | 38% |  |

*For the high risk immunocompromised, Blommaert et al. (2016b) mentions that there is no evidence of protection.

**Non-invasive pneumococcal disease**

For the non-invasive pneumococcal disease, KCE report considers 55% of the efficacy in IPD. This is quite optimistic considering that, contrary to PCV13, no study has proven a significant efficacy of PPV23 against all cause pneumonia (ambulatory or hospitalized).

For the high risk immunocompromised, Blommaert et al. (2016b) mentions that there is no evidence of any evidence of protection.

In Blommaert et al. (2016a), no efficacy was considered in the non-invasive pneumococcal disease, such as in Moro (2016).

**Duration of Benefits**

As recommended in the KCE report (Blommaert A. et al., 2016b), full efficacy is considered over the 1^st^ 2 years. Progressive decay is foreseen thereafter to be not any more effective as of year 5.

**7. SEROTYPE COVERAGE**

**7.1. Invasive Pneumococcal Disease**

Based on the detailed epidemiological data that we received from the reference surveillance laboratory, the following serotype coverages were used:

***Table 23: Vaccine serotypes coverage of IPD in the 50 y+***

| **Serotypes coverage** | **50 y.+** | **Source** |
| --- | --- | --- |
| **PCV13**    Proportion of PCV13 serogroups | 38.64% | Calculated based on data provided by Prof Verhaegen (based on 944 counts) |
| Proportion of vaccine serotypes in total IPD | **30.91%** | Calculated based on data provided by Prof Verhaegen (based on 944 counts) |
|  |  |  |
| **PPV23**  Proportion of PPV23 serogroups | 82.22% | Calculated based on data provided by Prof Verhaegen (based on 944 counts) |
| Proportion of vaccine serotypes in total IPD | **73.43%** | Calculated based on data provided by Prof Verhaegen (based on 944 counts) |

**7.2 Non-invasive Pneumococcal disease**

In the absence of data on serotype coverage for non-invasive CAP, we derived it from the ratio used by the KCE authors (Blommaert et al., 2016b), namely: 1.06 for PCV13 serotypes and 0.77 for PPV23 serotypes.

***Table 24: Vaccine serotypes coverage of non-invasive pneumococcal disease in the 50 y+***

| **Serotypes coverage** | **50 y.** | **Source** |
| --- | --- | --- |
| **PCV13** |  |  |
| Serotypes coverage | **32.9%** | Estimated based on ratio serotype coverage CAP/IPD in KCE report (Blommaert et al. 2016) |
| **PPV23**  Serotypes coverage | **56.7%** | Estimated based on ratio serotype coverage CAP/IPD in KCE report (Blommaert et al. 2016) |

**8. MEDICAL-CARE/VACCINATION COSTS**

Costs were derived from the same sources of information, namely a study done on a large sickfunds database and interviews (Beutels et al., 2006). These data were validated by Belgian experts during an Advisory Board on May 26, 2015. Participants included Bernard Vandercam (Infectious disease, UCL St Luc), Jan Verhaegen (KUL Leuven, Reference lab), Willy Peetermans (SHC, CAP review author) and Lieven Annemans (HECO specialist, UGent).

Costs reflect 2016 prices. The selected costs are associated with events modelled that are impactful and have an incremental difference between vaccination strategies (PCV13 vs. no vaccination/current situation).

The previous KCE reports (Beutels 2006 and Beutels 2011) and paper from Blommaert et al. (2016a) were used to estimate the cost of IPD and inpatient CAP. Due to the need for details per age group and public payer perspective (vs. health care perspective), we contacted Prof. Beutels and team to get these additional data.

As described in the previous KCE reports (Beutels 2006 and Beutels 2011), these cost data were derived from an intensive national face-to-face survey by members of the Christian Sickfunds with **pneumococcus isolation** (total of 915 patients). The healthcare costs related to a hospitalization were not limited to the costs at hospital. No detailed information was provided on the outpatient pneumonia.

For data missing in some age groups, we assumed values were equal to the age group immediately preceding it. The event costs are detailed in the table below.

Table: RIZIV/INAMI clinical event hospitalization costs

| **Diagnosis** | **Age groups** | **N** | **mean** |
| --- | --- | --- | --- |
| Meningitis | 65-74 y. | 2 | € 9681 |
|  | 75-85 y. | *Est.* | *€ 9681* |
| Bacteremia | 65-74 y. | 10 | € 13 251 |
|  | 75-84 y. | 11 | € 14 751 |
| Pneumonia | 65-75 y. | 17 | € 8 145 |
|  | 75-85 y. | 34 | € 15 554 |

*Source: UIA, Christian Sickfunds*

We did not have sufficient data to see the split per risk group. We therefore have used relative risk ratios as observed in the US to allocate age-specific values across risk groups within that age group (Weycker 2016). This is the only study that we have found on this relative risk ratio.

**8.1. Invasive Pneumococcal Disease**

***Table 25: Relative risk ratios in meningitis***

|  |  | **Relative Risk Ratios** | | | | | | | |
| --- | --- | --- | --- | --- | --- | --- | --- | --- | --- |
| **Age Groups** |  | **Low** | | **Moderate** | | | | **High** | |
| 65-74 y. |  | 1.00 | | 1.31 | | | | 1.05 | |
| 75-84 y. |  | 1.00 | | 1.31 | | | | 1.05 | |
| 85 y.+ |  | 1.00 | | 1.31 | | | | 1.05 | |
| ***Source:*** *Weycker 2016* | | | | | | | | | |
|  |  | |  | | |  | | |  |
| ***Table 26: Extrapolation of age-specific medical costs due to meningitis to age and risk-specific costs*** | | | | | | | | | |
|  | **Medical Care Costs** | | | | | | | | |
| **Age Groups** | **All Risk Groups** | | **Low Risk** | | **Moderate Risk** | | **High Risk** | | |
| 65-74 y. | € 9 681 | | € 8,598 | | € 11,263 | | € 9,005 | | |
| 75-84 y. | € 9 681 | | € 8,336 | | € 10,920 | | € 8,730 | | |
| 85 y+ | € 9 681 | | € 8,278 | | € 10,845 | | € 8,670 | | |

*Source : UIA, Christian Sickfunds*

***Table 27: Relative risk ratios in bacteraemia***

|  |  | **Relative Risk Ratios** | | | | | | | |
| --- | --- | --- | --- | --- | --- | --- | --- | --- | --- |
| **Age Groups** |  | **Low** | | **Moderate** | | | **High** | | |
| 65-74 y. |  | 1.00 | | 1.31 | | | 1.05 | | |
| 75-84 y. |  | 1.00 | | 1.31 | | | 1.05 | | |
| 85 y.+ |  | 1.00 | | 1.31 | | | 1.05 | | |
| ***Source:*** *Weycker, 2016* | | | | | | | | | |
|  |  | |  | | |  | | |  |
| ***Table 28 : Extrapolation of age-specific medical costs due to bacteraemia to age and risk-specific costs*** | | | | | | | | | |
|  | **Medical Care Costs** | | | | | | | | |
| **Age Groups** | **All Risk Groups** | | **Low Risk** | | **Moderate Risk** | | | **High Risk** | |
| 65-74 y. | € 13 251 | | € 11,768 | | € 15,416 | | | € 12,325 | |
| 75-84 y. | € 14 751 | | € 12,701 | | € 16,639 | | | € 13,303 | |
| 85 y.+ | € 14 751 | | € 12,614 | | € 16,525 | | | € 13,211 | |

*Source : UIA, Christian Sickfunds*

**8.2 Pneumococcal Pneumonia**

| ***Table 29 : Relative risk ratios in hospitalized pneumonia*** | | | | | | | |
| --- | --- | --- | --- | --- | --- | --- | --- |
|  |  | **Relative Risk Ratios** | | | | | |
| **Age Groups** |  | **Low** | **Moderate** | | **High** | | |
| 65-74 y. |  | 1.00 | 1.09 | | 1.20 | | |
| 75-84 y. |  | 1.00 | 1.09 | | 1.20 | | |
| 85 y.+ |  | 1.00 | 1.09 | | 1.20 | | |
| ***Source****: Weycker 2016* | | | | | | | |
|  |  |  | |  | | |  |
| ***Table 30: Extrapolation of age-specific medical costs due to inpatient pneumonia to age and risk-specific costs*** | | | | | | | |
|  | **Medical Care Costs** | | | | | | |
| **Age Groups** | **All Risk Groups** | **Low Risk** | **Moderate Risk** | | | **High Risk** | |
| 65-74 y. | € 8 145 | € 7,800 | € 8,501 | | | € 9,351 | |
| 75-84 y. | € 15 554 | € 14,747 | € 16,073 | | | € 17,679 | |
| 85 y.+ | € 14,963 | € 14,204 | € 15,482 | | | € 17,029 | |

*Source : UIA, Christian Sickfunds*

For outpatient pneumonia, the KCE report refers to a cost of €713 (year 2006 among patients ≥ 5 years). In 2016, this has been inflated to €858 (http://statbel.fgov.be). This is the only source of information on the aggregated cost for outpatient pneumonia.

***Table 31: Relative risk ratios in outpatients***

|  |  | **Relative Risk Ratios** | | | | | | | |
| --- | --- | --- | --- | --- | --- | --- | --- | --- | --- |
| **Age Groups** |  | **Low** | | | **Moderate** | | **High** | | |
| 65-74 y. |  | 1.00 | | | 1.03 | | 1.17 | | |
| 75-84 y. |  | 1.00 | | | 1.03 | | 1.17 | | |
| 85 y.+ |  | 1.00 | | | 1.03 | | 1.17 | | |
| *Source: Weycker et al., 2016* | | | | | | | | | |
|  |  | | |  | |  | | |  |
| ***Table 32: Extrapolation of age-specific medical costs due to outpatient pneumonia to age and risk-specific costs*** | | | | | | | | | |
|  | **Medical Care Costs** | | | | | | | | |
| **Age Groups** | **All Risk Groups** | | **Low Risk** | | **Moderate Risk** | | | **High Risk** | |
| 65-74 y. | € 858 | | € 841 | | € 868 | | | € 986 | |
| 75-84 y. | € 858 | | € 838 | | € 865 | | | € 982 | |
| 85 y.+ | € 858 | | € 814 | | € 841 | | | € 954 | |

*Source : UIA, Christian Sickfunds*

**8.3. Vaccine Costs**

**Table 33**: PCV13 Cost

|  |  | **INAMI Price***  **Per dose** | **Source** |
| --- | --- | --- | --- |
| PREVENAR13 (PCV13) |  | €63.64 | Pfizer |

**INAMI cost, considering ordinary beneficiaries (cfr KCE guidelines). Public price of 75.44€-11.8€ co-payment*

PCV13 may be administered during a routine visit to a GP or in combination with influenza vaccination or in a separate visit to a GP. We have, in the base case, considered that PCV13 will be administered at the same time as the inactivated influenza vaccine. This is justified by the fact that the inactivated influenza vaccines are also reimbursed in PCV13 proposed target population. We have consequently considered 50% of the cost for a visit to the GP (accredited, with Global Medical File), namely €10.24 (€20.48/2). This approach has also been considered in the KCE report (Blommaert 2016b).

Reimbursement of PPV23 has been rejected. As this paper considers the public payer’s perspective, no cost will be considered for PPV23.

**REFERENCES:**

Agency for Healthcare Research and Quality. Healthcare Cost and Utilization Project (HCUP) Nationwide Inpatient Sample (NIS), 2011. Rockville, MD. Available at: www.hcup-us.ahrq.gov/nisoverview.jsp. [Accessed June 2013]

Andrews NJ, Waight PA, George RC et al. Impact and effectiveness of 23-valent pneumococcal plysaccharide vaccine against invasive pneumococcal disease in the elderly in England and Wales . Vaccine. 2012 Nov 6;30 (48): 6802-8

Belgium National Health Survey 2013. Available at: https://s9xjb.wiv-isp.be/SASStoredProcess/guest?_program=/HISIA/SP/chrondis2013. [Accessed March 2015]

Beutels P, Blommaert A, Hanquet G, et al. Cost-effectiveness of 10- and 13-valent pneumococcal conjugate vaccines in childhood. Health Technology Assessment (HTA). Brussels: Belgian Health Care Knowledge Centre (KCE). 2011. Report 155 C. D/2011/10.273/21. Available at: https://kce.fgov.be/sites/default/files/page_documents/kce_155c_pneumococcal_vaccines.pdf. [Accessed March 2015]

Beutels P, Van Damme P., Oosterhuis-Kafeja F. Effects and costs of pneumococcal conjugate vaccination of Belgian children. Health Technology Assessment (HTA). Brussels: Belgian Health Care Knowledge Centre (KCE) : 2006. KCE reports 33C (D/2006/10.273/53)

Blommaert et al., Pneumonia in adults in Belgium, Poster presented at ECCMID, 11 March 2013

Blommaert A, Bilcke J, Willem L, Verhaegen J, Goossens H, Beutels P. [The cost-effectiveness of pneumococcal vaccination in healthy adults over 50: An exploration of influential factors for Belgium.](https://www.ncbi.nlm.nih.gov/pubmed/26988257)Vaccine. 2016 Apr 19;34(18):2106-12. doi: 10.1016/j.vaccine.2016.03.003

Blommaert A., Hanquet G, Willem L et al., Use of pneumococcal vaccines in the elderly: an economic evaluation. Health Technology Assessment (HTA) Brussels : Belgian Health Care Knowledge Centre (KCE). 2016. KCE reports 274. D/2016/10.273/79

Bhorat AE, Madhi SA, Laudat F, Sundaraiyer V, Gurtman A, Jansen KU, Scott DA, Emini EA, Gruber WC, Schmoele-Thoma B. Immunogenicity and safety of the 13-valent pneumococcal conjugate vaccine in HIV-infected individuals naive to pneumococcal vaccination. AIDS. 2015 Jul 17;29(11):1345-54

Bonten MJ, Huijts SM, Bolkenbaas M, et al. Polysaccharide Conjugate Vaccine against Pneumococcal Pneumonia in Adults. N Engl J Med 2015;372;12:1114-25. Doi : 10.1056/NEJMoa1408544

CBIP BCFI 2015. Répertoire Commenté des médicaments. Available at http://www.cbip.be/fr/start. [Accessed March 2015]

Cleemput I, Neyt M, Van de Sande S. Recommandations belges pour les évaluations économiques et les analyses d’impact budgétaire : deuxième édition, rapport KCE 183B, www.kce.fgov.be, 2012.

Cordonnier C, Ljungman P, Juergens C, Maertens J, Selleslag D, et al; 3003 Study Group. Immunogenicity, safety, and tolerability of 13-valent pneumococcal conjugate vaccine followed by 23-valent pneumococcal polysaccharide vaccine in recipients of allogeneic hematopoietic stem cell transplant aged ≥2 years: an open-label study. Clin Infect Dis. 2015 Aug 1;61(3):313-23

De Montalembert M, Abboud MR, Fiquet A, Inati A, Lebensburger JD, Kaddah N, et al . 13-valent pneumococcal conjugate vaccine (PCV13) is immunogenic and safe in children 6-17 years of age with sickle cell disease previously vaccinated with 23-valent pneumococcal polysaccharide vaccine (PPSV23): Results of a phase 3 study. Pediatr Blood Cancer. 2015 Aug;62(8):1427-36.

Flamaing J, De Backer W, Van Laethem Y, et al. Pneumococcal lower respiratory tract infections in adults: an observational case-control study in primary care in Belgium. BMC Fam Pract 2015;16:66. doi: 10.1186/s12875-015-0282-1.

Hak E, Grobbee DE, Sanders EA, et al. Rationale and design of CAPITA: a RCT of 13-valent conjugated pneumococcal vaccine efficacy among older adults. Neth J Med 2008;66:378-83.

Hung TY, Kotecha RS, Blyth CC, Steed SK, Thornton RB, Ryan AL, Cole CH, Richmond PC. Immunogenicity and safety of single-dose, 13-valent pneumococcal conjugate vaccine in pediatric and adolescent oncology patients. Cancer. 2017 Jul 11. doi: 10.1002/cncr.30764. [Epub ahead of print]

Institut National d'assurance Maladie-Invalidité. Soins de santé : Prix et honoraires. Available at: www.inami.fgov.be. [Accessed March 2015]

Institut Scientifique de Santé Publique. Epidémiologie. Available at: https://www.wiv-isp.be/Pedisurv/AnnualReports/2012/jaarverslag_2012_fr.pdf. [Accessed November 2014]

Institut Scientifique de Santé Publique. Epidémiologie. Available at: https://www.wiv-isp.be/Epidemio/epifr/plabfr/plabanfr/11_030f_r.pdf. [Accessed March 2015]

Jallow S, Madhi SA, Madimabe R, Sipambo N, Violari A, Kala U, Petersen K, Naidoo S, Verwey C, Moore DP, Nunes MC. Immunogenicity of 13-valent pneumococcal conjugate vaccine among children with underlying medical conditions. Vaccine. 2017 Aug 3;35(34):4321-4329.

Klugman KP, Madhi SA, Huebner RE, et al. A trial of a 9-valent pneumococcal conjugate vaccine in children with and those without HIV infection. N Engl J Med 2003;349:1341-8.

Lombardi F, Belmonti S, Fabbiani M, Morandi M, Rossetti B, Tordini G, Cauda R, De Luca A, Di Giambenedetto S, Montagnani F. Immunogenicity and Safety of the 13-Valent Pneumococcal Conjugate Vaccine versus the 23-Valent Polysaccharide Vaccine in Unvaccinated HIV-Infected Adults: A Pilot, Prospective Controlled Study. PLoS One. 2016 Jun 3;11(6):e0156523. doi: 10.1371/journal.pone.0156523. eCollection 2016.

Mangen MJ, Bonten MJ, de Wit GA. Rationale and design of the costs, health status and outcomes in community-acquired pneumonia (CHO-CAP) study in elderly persons hospitalized with CAP. BMC Inf Dis 2013;13:597.

Mangen MJ, Rozenbaum MH, Huijts SM, et al. Cost-effectiveness of adult pneumococcal conjugate vaccination in the Netherlands. Eur Respir J 2015;46(5):1407-16. DOI:10.1183/13993003.00325-2015.

Rodríguez González-Moro JM, Menéndez R, Campins M, Lwoff N, Oyagüez I, Echave M, Rejas J, Antoñanzas F., [Cost Effectiveness of the 13-Valent Pneumococcal Conjugate Vaccination Program in Chronic Obstructive Pulmonary Disease Patients Aged 50+ Years in Spain.](https://www.ncbi.nlm.nih.gov/pubmed/26547199)Clin Drug Investig. 2016 Jan;36(1):41-53. doi: 10.1007/s40261-015-0345-z.

National institute of Health and Welfare Finland. Incidence of invasive pneumococcal disease in Finland. Available at: <https://www.thl.fi/en/web/thlfi-en/research-and-expertwork/projects-and-programmes/monitoring-the-population-effectiveness-of-pneumococcal-conjugate-vaccination-in-the-finnish-national-vaccination-programme/incidence-of-invasive-pneumococcal-disease-in-finland> [Accessed February 2017]

Rodrigo C, Bewick T, Sheppard C, et al. Impact of infant 13-valent pneumococcal conjugate vaccine on serotypes in adult pneumonia. Eur Respir J 2015;45:1632-41. Doi: 10.1183/09031936.00183614

Salo H, Sintonen H, Nuorti JP, et al. Economic evaluation of pneumococcal conjugate vaccination in Finland. Scand J Infect Dis 2005;37(11-12):821-32.

Sisk JE, Whang W, Butler JC, et al. Cost-effectiveness of vaccination against invasive pneumococcal disease among people 50 through 64 years of age: role of comorbid conditions and race. Ann Intern Med 2003;138(12):960-8.

Smith KJ, Zimmerman RK, Lin CJ, et al. Alternative strategies for adult pneumococcal polysaccharide vaccination: a cost-effective analysis. Vaccine 2008;26:1420-31.

SPF Economie. Population - Population au 1er janvier et par âge 2015. 2013. Available at: http://statbel.fgov.be/. [Accessed February 2017]

SPF Economie. Statistics Belgium – Mortalité, espérance de vie et causes de décès. 2015. Available at: http://statbel.fgov.be/fr/statistiques/chiffres/population/deces_mort_esp_vie/. [Accessed February 2017]

Stirbu-Wagner I, Dorsman SA, Visscher S, Davids R. Facts and numbers in primary care. The Netherlands Information Network of General Practice. 2010. Utrecht/Nijmegen. NIVEL/IQ.16-2.2013

Suaya JA, Jiang Q, Scott DA, Gruber WC, Webber C, Schmoele-Thoma B, Hall-Murray CK, Jodar L, Isturiz RE. Post hoc analysis of the efficacy of the 13-valent pneumococcal conjugate vaccine against vaccine-type community-acquired pneumonia in at-risk older adults. Vaccine. 2018; 36(11): 1477-1483.

Torres A., Blasi F., Peetermans W.E. et al., The aetiology and antibiotic management of community-acquired pneumonia in adults in Europe : a literature review, Eur J Clin Microbiol Infect Dis (2014) 33:1065-1079.doi 10.1007/s10096-014-2067-1

Truyers C, Goderis G, Dewitte H, et al. The Intego database: background, methods and basic results of a Flemish general practice-based continuous morbidity registration project. BMC Med Inform Decis Mak 2014;14:48. doi: 10.1186/1472-6947-14-48.

van Hoek AJ, Andrews N, Waight PA, et al. The effect of underlying clinical conditions on the risk of developing invasive pneumococcal disease in England. J Infect 2012;65(1):17-24.

van Werkhoven CH, Huijts SM, Bolkenbaas M, et al. Herd effects of infant immunisation with pneumococcal conjugate vaccines. A post-hoc analysis of the CAP-pilot study and Community-Acquired Pneumonia immunisation Trial in Adults (CAPiTA), poster presented at ECCMID congress on 13 April 2015.

van Werkhoven CH, Huijts SM, Bolkenbaas M, Grobbee DE, Bonten MJ. [The Impact of Age on the Efficacy of 13-valent Pneumococcal Conjugate Vaccine in Elderly.](https://www.ncbi.nlm.nih.gov/pubmed/26265498)Clin Infect Dis. 2015 Dec 15;61(12):1835-8.

Verhaegen J, Flamaing J, De Backer W, et al. Epidemiology and outcome of invasive pneumococcal disease among adults in Belgium, 2009-2011. Euro Surveill 2014;19(31):14-22. Available at: http://www.eurosurveillance.org/ViewArticle.aspx?ArticleId=20869

Welte T, Torres A, Nathwani D. Clinical and economic burden of community-acquired pneumonia among adults in Europe. Thorax 2012;67:71-79. Doi : 10.1136/thx.2009.129502

Weycker D, Sato R, Strutton D, Edelsberg J, Atwood M, Jackson L. Public Health and Economic Impact of 13-Valent Pneumococcal Conjugate Vaccine in US Adults Aged ≥50 Years, *Vaccine* 2012;30:5437-44

Weycker D, Farkouh R, Strutton D, et al. Rates and costs of invasive pneumococcal disease and pneumonia in persons with underlying medical conditions. BMC Health Serv Res. 2016; 16:182. Doi 10.1186/s12913-016-1432-4

**S1 ONLINE SUPPLEMENT FOR Cost-Effectiveness of PCV13 in**

**Moderate/High-Risk Belgian Adults Aged 65-84 Years:**

**Results from Sensitivity Analyses**


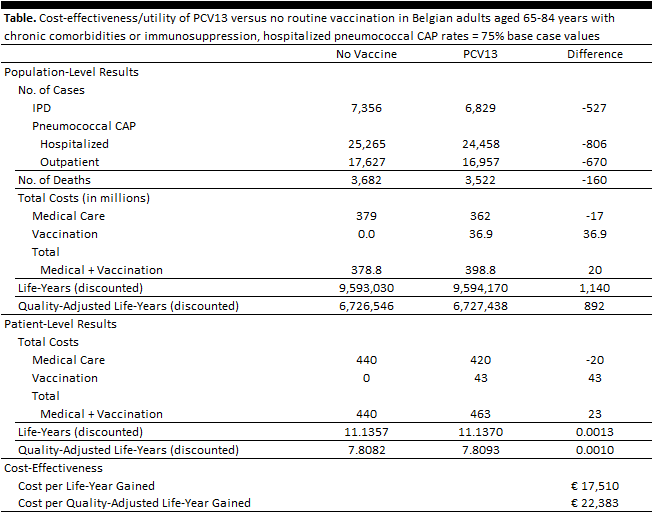


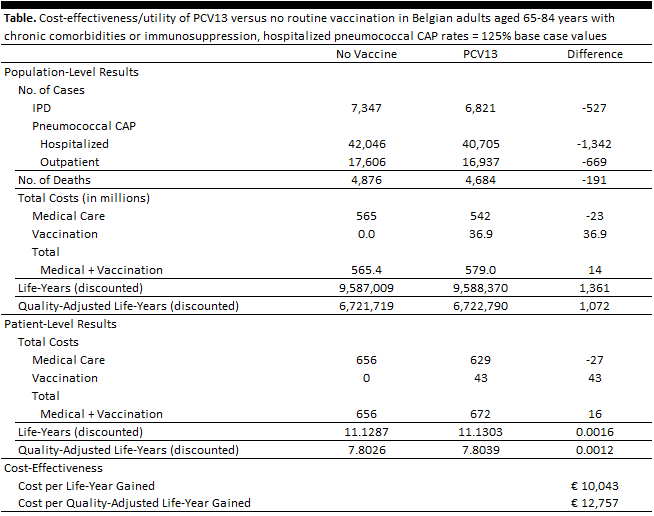


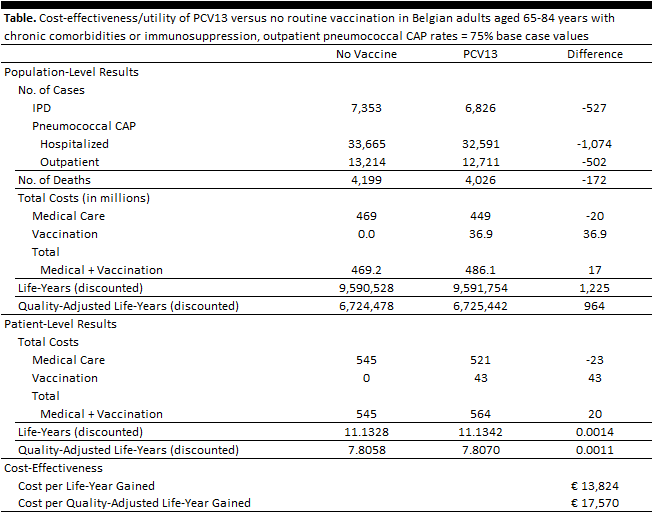


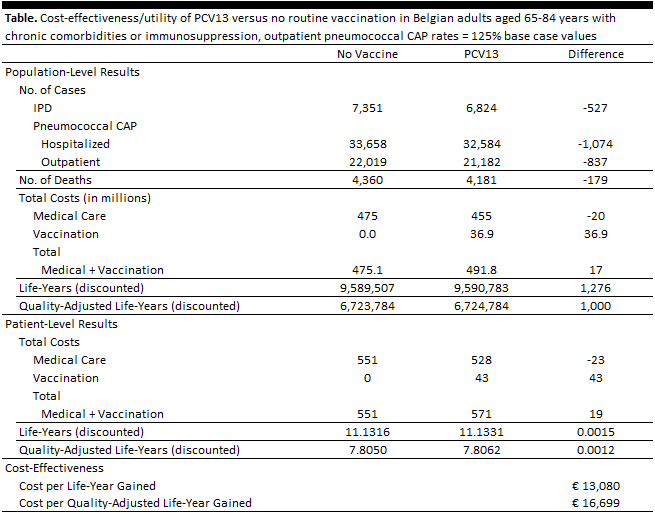


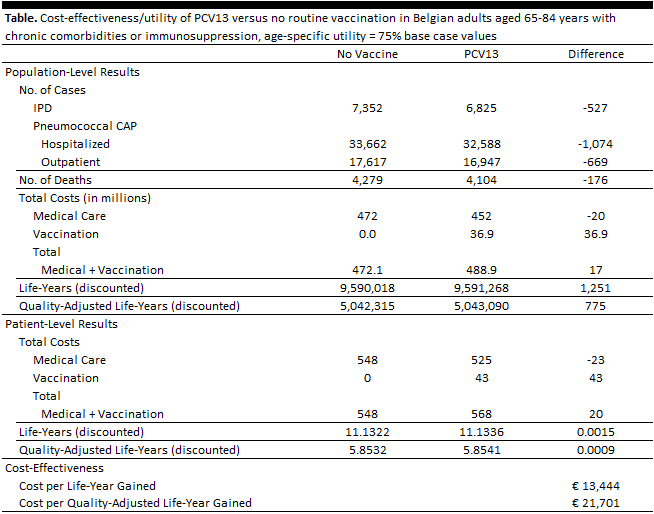


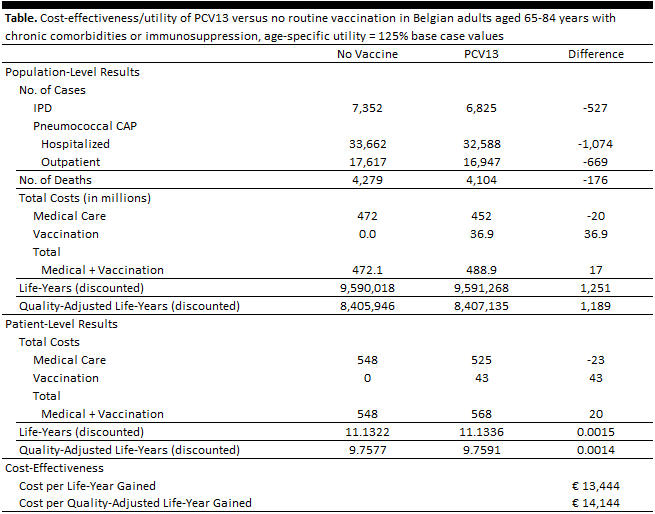


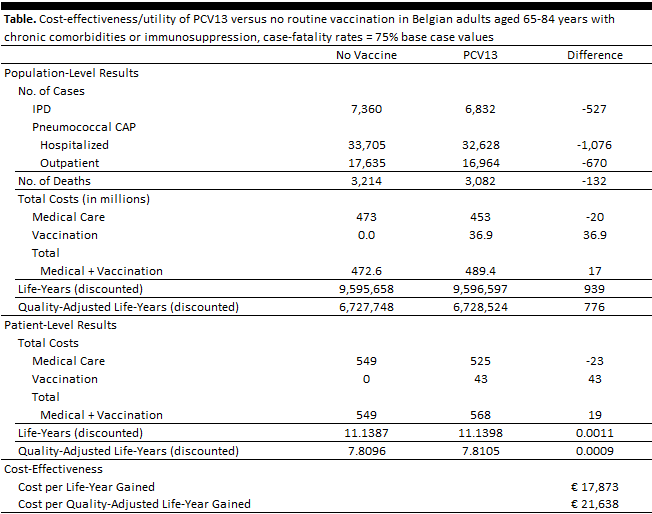


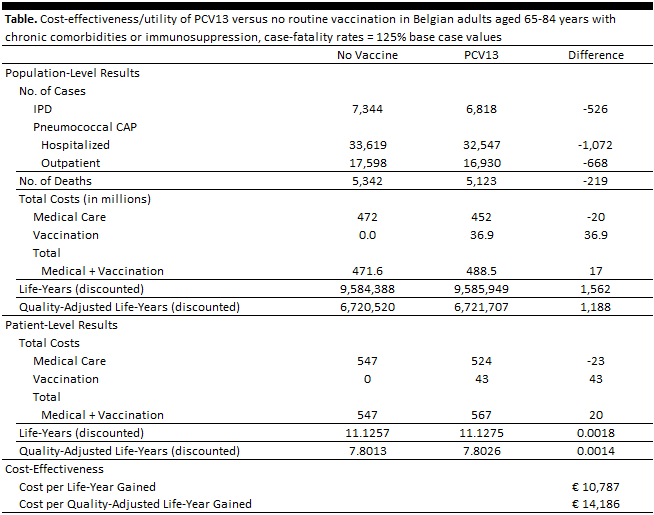


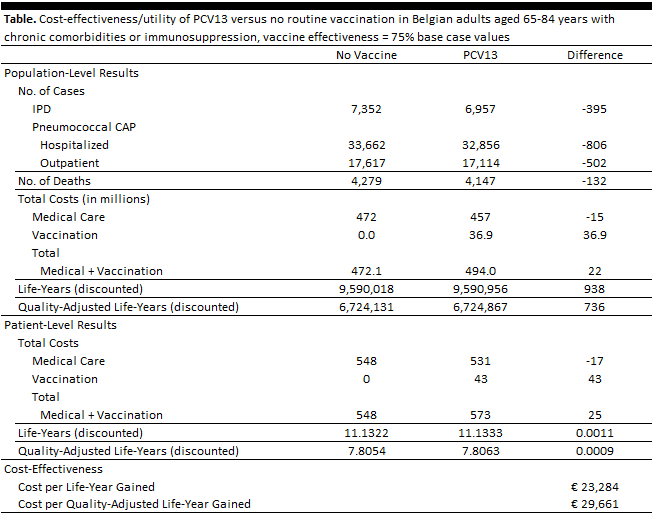


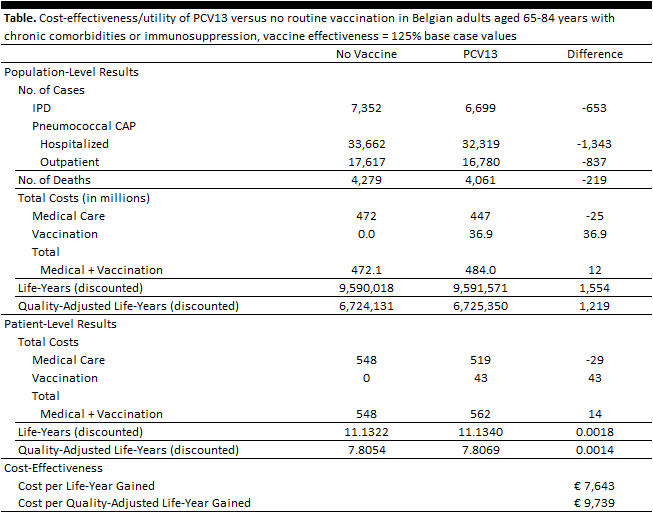


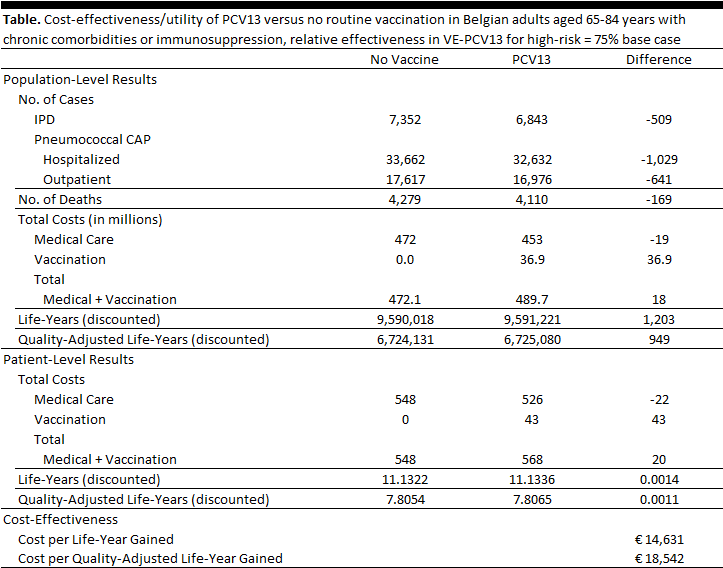


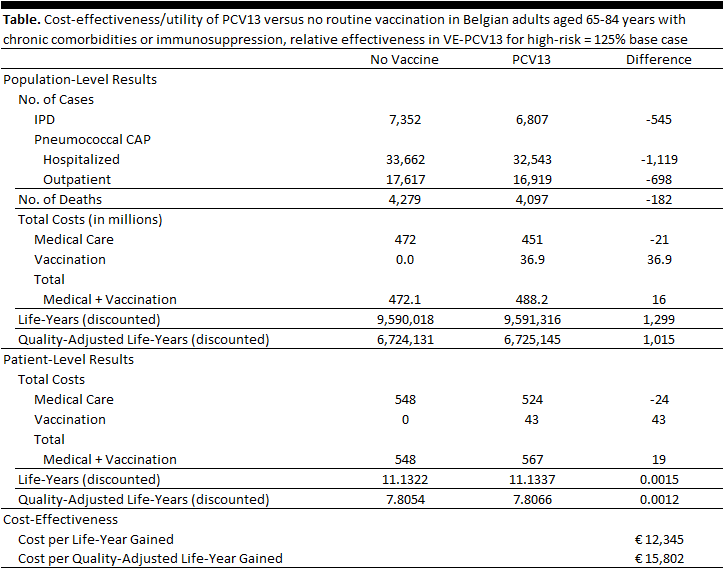


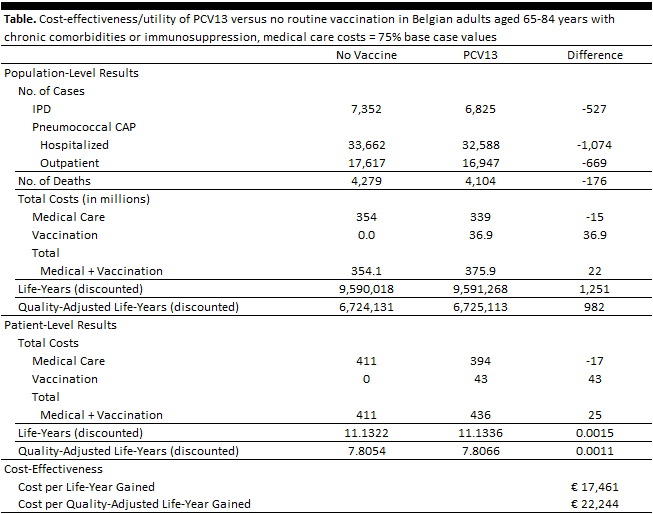


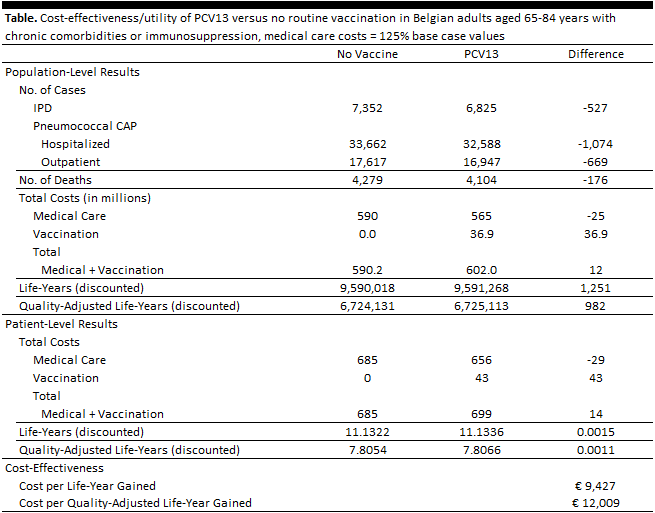


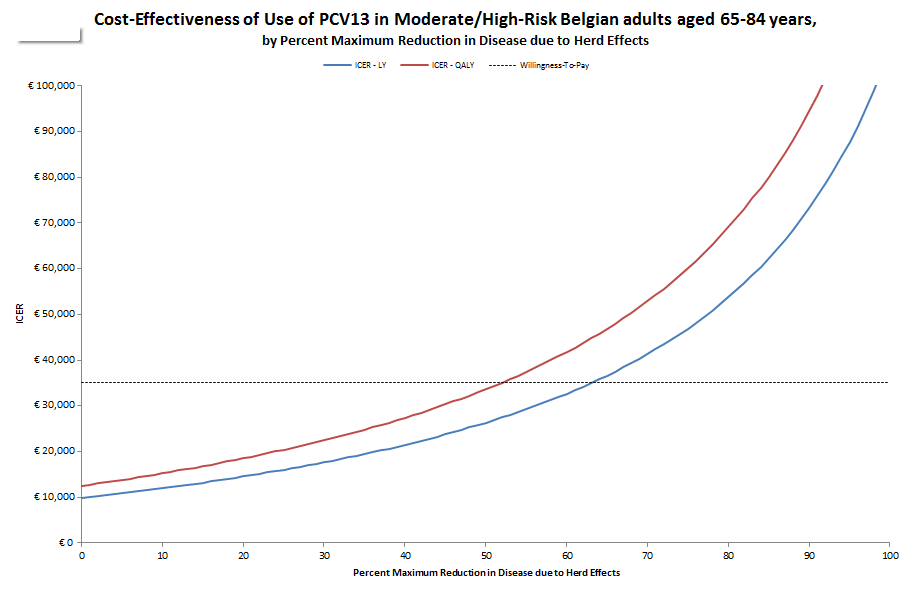


**S1 ONLINE SUPPLEMENT FOR Cost-Effectiveness of PCV13 in**

**Moderate/High-Risk Belgian Adults Aged 65-84 Years:**

**Results from Scenario Analyses**


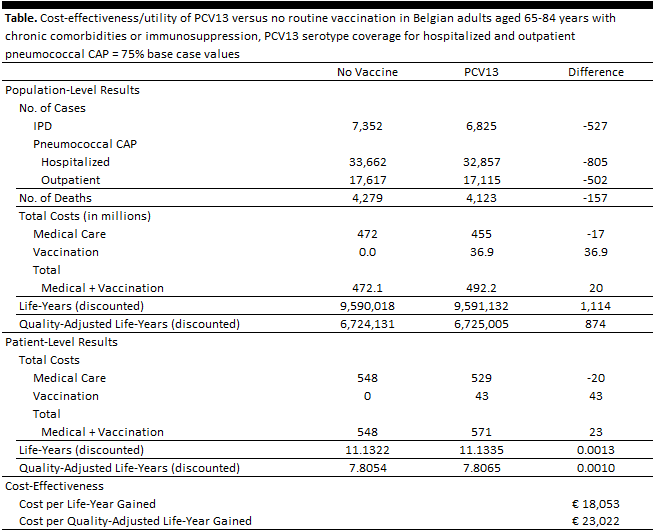


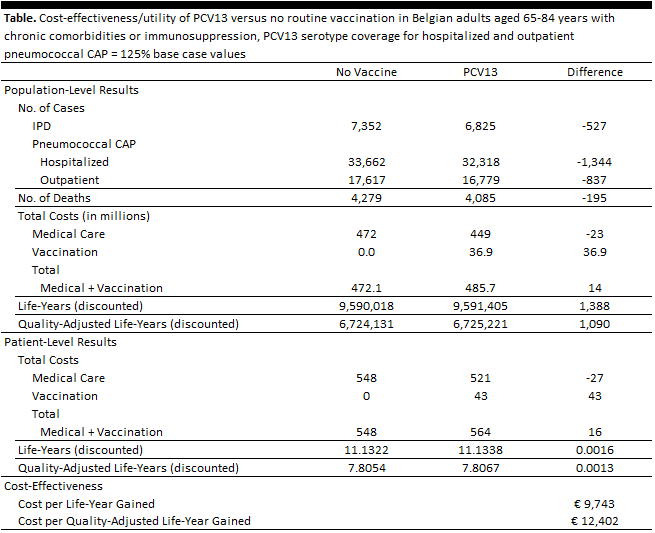


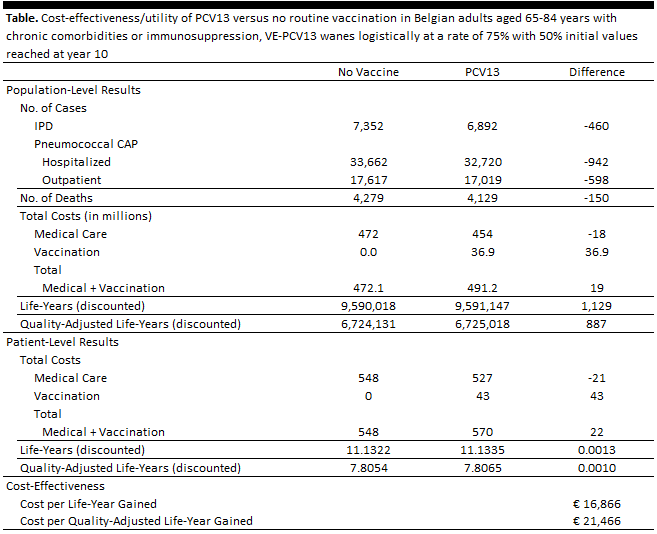


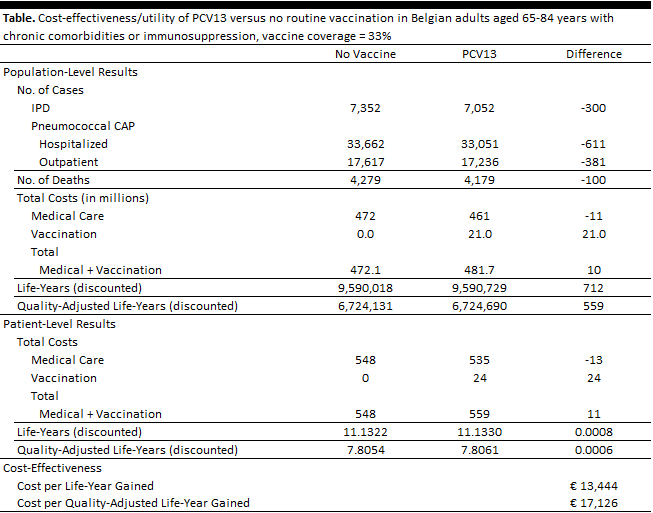


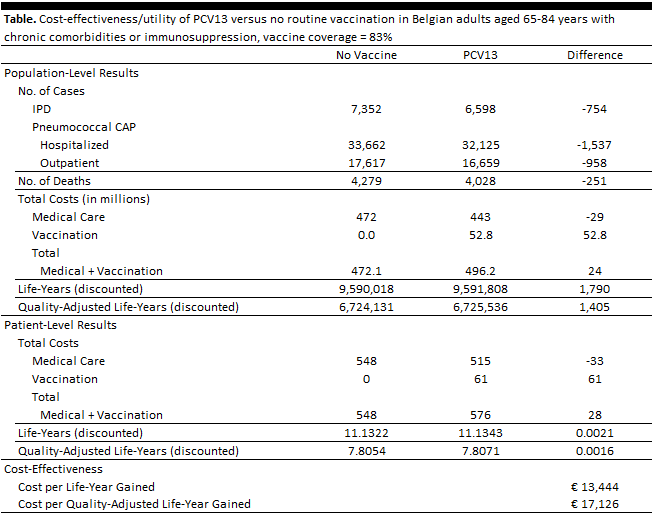


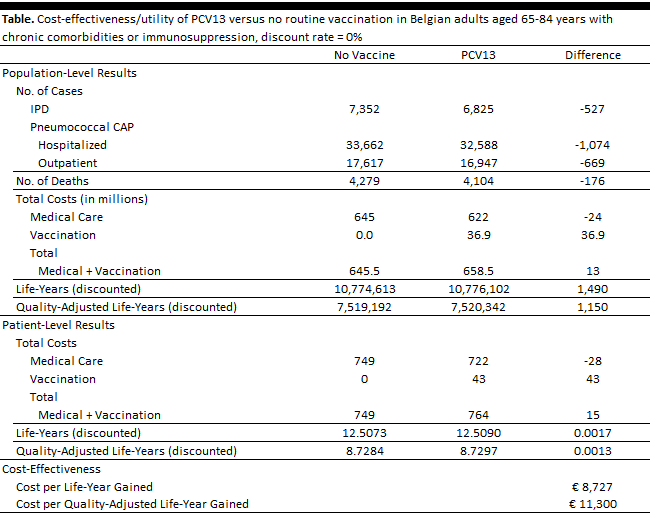


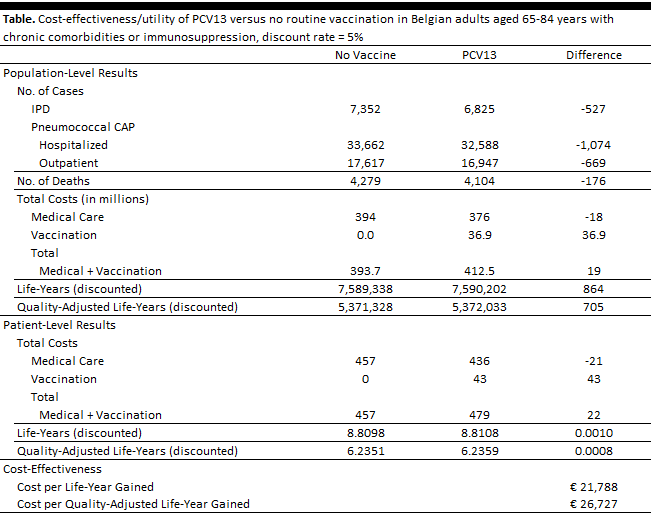


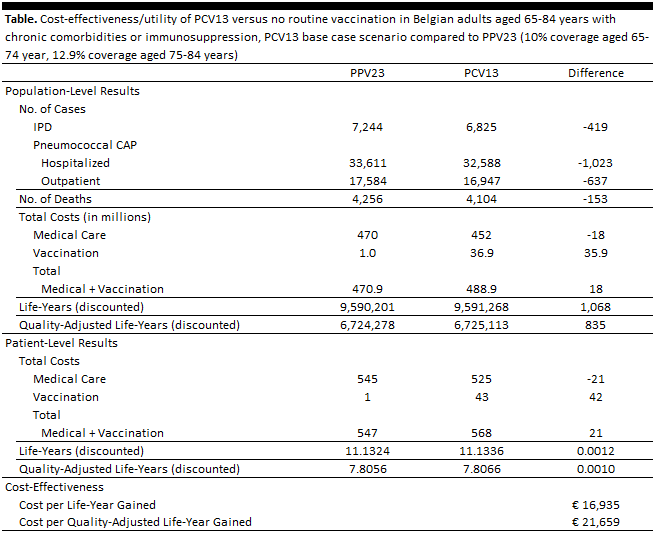


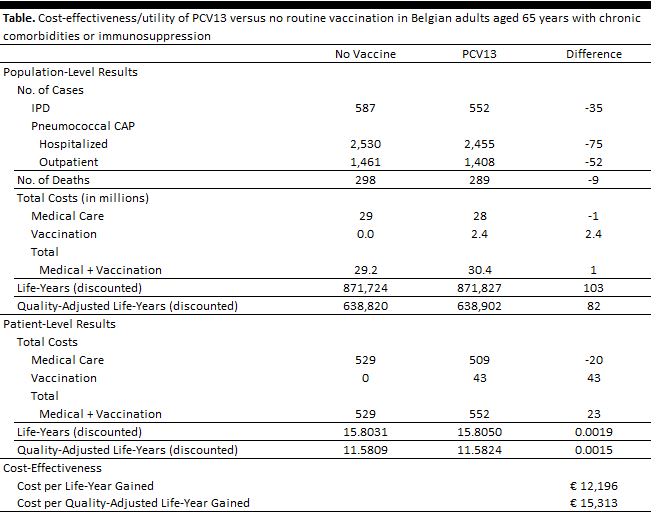

Supplement: S1 File — (DOC) [file pone.0199427.s001.doc]
